# Natural Products as Novel Neuroprotective Agents; Computational Predictions of the Molecular Targets, ADME Properties, and Safety Profile

**DOI:** 10.3390/plants11040549

**Published:** 2022-02-18

**Authors:** Sahar Saleh Alghamdi, Rasha Saad Suliman, Norah Abdulaziz Aljammaz, Khawla Mohammed Kahtani, Dimah Abdulqader Aljatli, Ghadeer M. Albadrani

**Affiliations:** 1 College of Pharmacy, King Saud bin Abdulaziz University for Health Sciences, Riyadh 11481, Saudi Arabia; sulimanr@ksau-hs.edu.sa (R.S.S.); aljammaz369@ksau-hs.edu.sa (N.A.A.); kahtani085@ksau-hs.edu.sa (K.M.K.); aljatli097@ksau-hs.edu.sa (D.A.A.); 2 King Abdullah International Medical Research Centre (KAIMRC), Ministry of National Guard Health Affairs, Riyadh 11481, Saudi Arabia; 3 Department of Biology, College of Science, Princess Nourah bint Abdulrahman University, Riyadh 11474, Saudi Arabia; gmalbadrani@pnu.edu.sa

**Keywords:** medicinal plants, neurological diseases, microglia polarization, neuroinflammation, ADME, target production, immune response

## Abstract

Neurodegenerative diseases (NDs) are one of the most challenging public health issues. Despite tremendous advances in our understanding of NDs, little progress has been made in establishing effective treatments. Natural products may have enormous potential in preventing and treating NDs by targeting microglia; yet, there have been several clinical concerns about their usage, primarily due to a lack of scientific evidence for their efficacy, molecular targets, physicochemical properties, and safety. To solve this problem, the secondary bioactive metabolites derived from neuroprotective medicinal plants were identified and selected for computational predictions for anti-inflammatory activity, possible molecular targets, physicochemical properties, and safety evaluation using PASS online, Molinspiration, SwissADME, and ProTox-II, respectively. Most of the phytochemicals were active as anti-inflammatory agents as predicted using the PASS online webserver. Moreover, the molecular target predictions for some phytochemicals were similar to the reported experimental targets. Moreover, the phytochemicals that did not violate important physicochemical properties, including blood-brain barrier penetration, GI absorption, molecular weight, and lipophilicity, were selected for further safety evaluation. After screening 54 neuroprotective phytochemicals, our findings suggest that Aromatic-turmerone, Apocynin, and Matrine are the most promising compounds that could be considered when designing novel neuroprotective agents to treat neurodegenerative diseases via modulating microglial polarization.

## 1. Introduction

Once the body is exposed to damage caused by external or internal harmful stimuli, the immune system will defend against these threats and initiate the repairing process [1,2]. After recognition of foreign agents, inflammatory processes will begin where many inflammatory mediators are released, such as tumor necrosis factor-α (TNF-α), interleukins (ILs), leukotrienes, nitric oxide (NO), and prostaglandin E2 (PGE2), besides the activation of inflammatory pathways such as nuclear factor-kappa-B (NF-κB), mitogen-activated protein kinase (MAPK), and Janus kinase signal transducer and activator of transcription (JAK/STAT) to minimize the impending of the damage [1]. After that, inflammation resolution is mediated by reducing mediators’ production, which leads to diluting the chemokine gradients and reducing the white blood cells (WBC) sensation at the site of damage. Although this biological response, inflammation, is a vital defensive mechanism of the body, especially in acute conditions, it also plays a significant role in several pathophysiological disorders [3,4]. If the resolution process fails and the inflammatory response continues, it may progress into persistent and chronic inflammation, as the excess production of cytokines and inflammatory mediators is associated with many neurodegeneration diseases [5,6,7].

Neurodegeneration Diseases (NDs) is a phrase that refers to the loss of neurons in diseases of the central nervous system such as Alzheimer’s disease (AD), Parkinson’s disease (PD), and Multiple sclerosis (MS). More recent attention has focused on the role of microglia-mediated inflammatory singling in the onset and progression of neurodegenerative disease [8]. The polarization of activated microglia into the M1 phenotype has been linked to the release of pro-inflammatory mediators that promote neuroinflammation and neuronal damage [9]. The interest that activated microglia contributes to the progression of chronic neurodegeneration was first postulated in brain samples of AD patients [10]. Studies showed an extracellular deposition of the protein amyloid-beta [Aβ]-containing plaques and the development of intracellular neurofibrillary tangles (NFT) composed of hyper-phosphorylated tau proteins [11,12]. Upon the accumulation of Aβ, microglia are activated as phagocytic cells and are believed to clear Aβ deposits initially; however, as the disease progresses, microglia produce pro-inflammatory mediators and reactive oxygen species (ROS), as well as lose their ability to clear Aβ, promoting neuronal degeneration and disease progression [13]. Moreover, pro-inflammatory microglia have exacerbated tau pathology by increasing its phosphorylation [14]. In the case of PD, studies reported the accumulation of Lewy bodies, which are intracellular inclusions containing α-synuclein, as well as the loss of dopaminergic neurons in the substantia nigra, which are the hallmarks of PD [15,16]. Microglial cells have been observed to be gradually activated in the substantia nigra of PD patients [17]. Moreover, in early PD, the degree of microglial activity was linked to dopaminergic terminal loss [18]. Additionally, MS is characterized by neuroaxonal degeneration, which results in irreversible neurological impairment [19]. Microglia have been shown to play a direct role in the progression of MS, in which pro-inflammatory mediators produced by activated microglia contribute to myelin destruction [20,21].

Microglia are specialized innate immune cells that function in the brain in place of macrophages. It maintains the central nervous system’s homeostasis by regulating two cycles classified into M1 and M2 based on their metabolism and secretory mediators [22,23,24]. M1 is the pro-inflammatory phase induced by interferon-gamma combined with lipopolysaccharide, INF-γ/LPS, resulting in the production of mediators such as IL-1β, IL-6, IL-12, IL-18, and IL-23, as well as TNF-α, which cause neuronal damage [24,25]. M2, on the contrary, is an anti-inflammatory phase that is triggered by, but not limited to, Toll-like receptors agonists (TLRs agonists), Transforming growth factor-beta (TGF-β), and glucocorticoids, resulting in the release of mediators such as interleukins IL-4, IL10, and IL-13, as well as Arginase-1 (ARG1), which relieve inflammatory responses and enhance neuronal repair [24,25]. Hence, suppressing inflammatory responses via targeting the microglia is a promising approach in managing neuroinflammatory-based diseases. In this context, several natural products have such properties and may influence the prevention, incidence, and severity of neurodegenerative illness.

Only palliative treatments are available for these neurodegenerative disorders, none of which can appreciably slow or cure the underlying cause [26]. Therefore, new treatments and novel therapeutic approaches are urgently needed; regulation of microglial polarization from M1 to M2 phenotypes seems to be a viable strategy for NDs treatment and prevention. As per the World Health Organization (WHO), neurodegenerative illnesses that affect motor function are estimated to become the second-leading cause of mortality in the next 20 years [27]. Thus, in this study, we aspire to shed some insight into phytochemical compounds used to treat neurodegenerative diseases such as Alzheimer’s disease (AD), Parkinson’s disease (PD), and Multiple sclerosis (MS) by investigating their pharmacokinetic properties, predicting their biological targets, assessing their safety/toxicity profiles, and cytochrome enzyme inhibition using computational techniques.

## 2. Study Design

**Figure 1:**
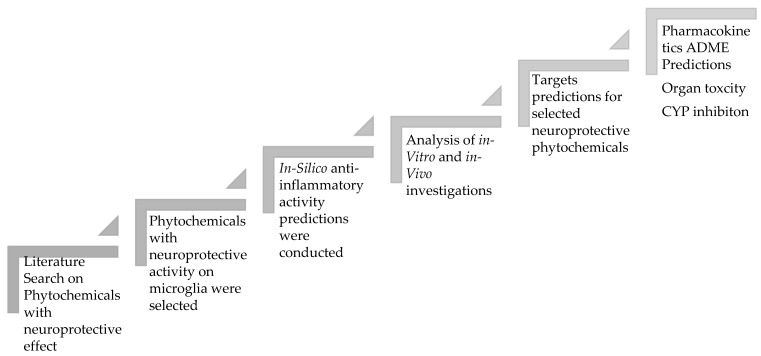
The steps involved in the study design of neuroprotective phytochemicals.

Below is the study design that involves several steps, as shown in Figure 1.

## 3. Results

### 3.1. Proposed Mechanisms Involved in the Neuroprotective Effects of Phytochemicals in Neurodegenerative Diseases Based on the Reported Literature

#### 3.1.1. AD

**Figure 2:**
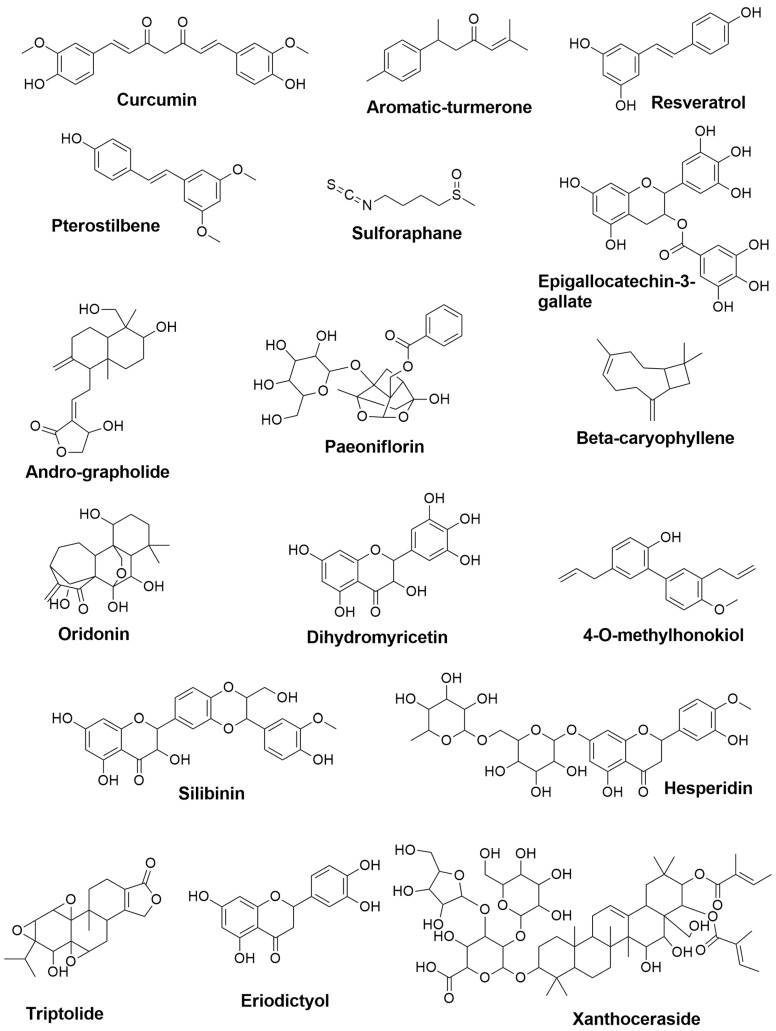

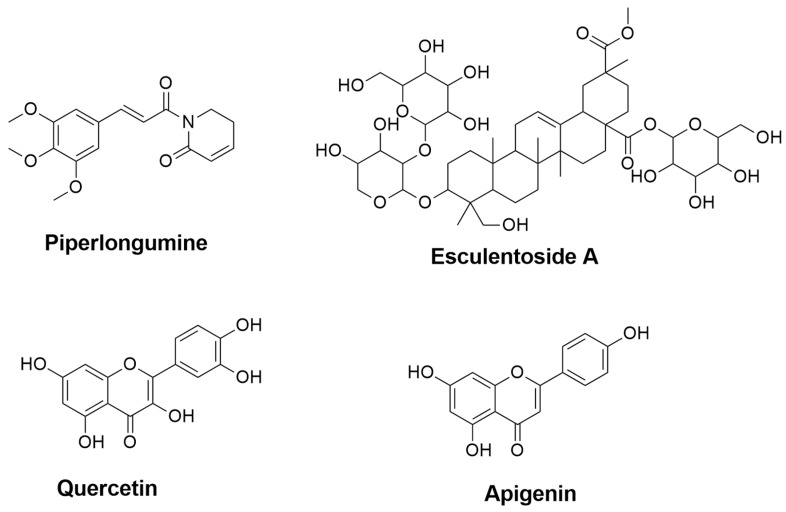
The 2D chemical structures of the neuroprotective phytochemical used for AD treatments.

The prevalence of AD greatly rises with age [28], and in 1997, approximately 2.32 million people in the United States had Alzheimer’s disease, and by 2047, it is expected that 8.64 million individuals will be diagnosed with AD, resulting in a massive societal and economic burden [29]. Although no treatments are available to stabilize or reverse the neurodegenerative process, several palliative disease-modifying medicines are now in development with early clinical investigations [30]. Natural products are a viable treatment option. A wide range of phytochemical compounds and secondary bioactive metabolites has been studied pre-clinically and clinically to prevent and attenuate the multifactorial pathologies of AD (chemical structures are summarized in Figure 2) via microglial modulation.

In the case of physiological conditions, microglia’s number and functions are tightly regulated. Nonetheless, if stimuli bind to the pattern-recognition receptors [PRRs] on the surface of microglia [31], microglia will be over-activated to respond to the insult through shifting into different functional states, modifying its proliferation, morphology, phagocytic activity, antigen presentation, and the production of inflammatory markers such as cytokines and chemokines [32]. The process involves a diverse set of signaling pathways, including but not limited to tumor necrosis factors (TNFs), interferons (IFNs), chemokines, colony-stimulating factors (CSFs), and interleukins (ILs) [33]. This sustained over-activation of microglia has been observed in various neurodegenerative diseases, and targeting these pathways is one of the proposed mechanisms of multiple phytochemical compounds, as discussed in detail below.

##### Pattern Recognition Receptors (PRRs)

Pattern recognition receptors (PRRs) are present on the plasma membrane of microglia that are capable of detecting foreign bodies that stimulate microglia. PRR subfamilies that are predominantly expressed by microglia include toll-like receptors (TLR), inflammasome-forming nucleotide-binding oligomerization domain (nod)-like receptors (NLRs), triggering receptor expressed on myeloid cells (TREMs), and other receptors [34]. Inflammatory factors such as IL-1β, IL-6, TNF-α, ROS, and Cyclooxygenase-2 (COX-2) are produced due to the interaction between the ligand and PRR receptor, as well as boosting microglial phagocytic activity in the short term microglial activation. However, chronic activation will impair this protective mechanism and might exacerbate neurodegeneration [34]. TLR4 signaling pathways, for example, are activated in microglia during neuroinflammation, resulting in caspase-8 and caspase-3 activation, nuclear translocation of NF-κB, and expression of genes implicated in the inflammatory response; inhibiting TLR4 activation and signaling is thus a beneficial mechanism.

For instance, Eriodictyol, a natural flavonoid found in citrus fruits and peanuts, has been shown to alleviate neuroinflammation, amyloidogenesis, and memory impairment induced by Lipopolysaccharide (LPS) through many mechanisms, one of which is via inhibiting TLR4 activation [35]. Furthermore, NLRP3, which belongs to the NOD-like receptors (NLRs) family, is another target of Esculentoside A and Pterostilbene according to in-vitro models, where they inhibit the Aβ_1−42_ induced NLRP3/caspase-1 inflammasome in BV-2 cells, as shown in Table 1 [36,37].

##### Transcription Factors (TFs)

Transcription factors are proteins that are involved in the regulation of the expression of genes. NF-κB represents a family of transcription factors that control the expression of a variety of genes involved in cell death, inflammation, proliferation, and differentiation [78]. Multiple studies have revealed that NF-κB is activated in several NDs and engaged in microglia-mediated Aβ toxicity, making it one of the most important transcription factors for the expressions of pro-inflammatory cytokines [79]. The activation of NF-κB results in the phosphorylation of NF-κB inhibitor, IκB, via the IκB kinase (IKK) signalosome complex leading to transcription of pro-inflammatory mediators, such as iNOS, COX-2, TNF-α, and IL-1β [80,81] Therefore, inhibiting the NF-κB will suppress the release of these inflammatory markers, which is a mechanism of a variety of natural plants, such as Piperlongumine, Aromatic-turmerone, Oridonin, and Andrographolide, as demonstrated in pre-clinical studies that shown in Table 1. Epigallocatechin-3-gallate, a polyphenolic compound found in green tea, has been shown to suppress the expression of TNFα, Il- β, Il-6, and iNOS in Aβ-stimulated EOC 13.31 mouse immortalized microglial cells [49]. It is worth noting that a phase III clinical trial for Epigallocatechin-3-gallate is being conducted to treat the early stages of Alzheimer’s disease; however, the results have not yet been published [82].

Moreover, signal transducer and activator of transcription (STATs), another family of the transcription factors that expressed and mediated various functions, including proliferation, apoptosis, and differentiation in response to cytokines [83]. STAT1 is assumed to be a key signaling regulator via IFNs involved in innate immune responses, including type I and type II IFNs [84]. STAT3, on the other hand, mediates the cells’ survival and proliferation of the IL-6 through regulating the expression of genes involved in the cell cycle and suppression of apoptosis [84]. STAT proteins are phosphorylated by the Janus kinase family, which includes JAK1, JAK2, and TYK2, causing them to translocate to the nucleus and stimulate transcription of their target genes. The abnormal activation of JAK/STAT signaling in innate immune cells has been linked to AD and MS [84].

Resveratrol, a naturally occurring dietary polyphenolic compound found in abundance in the skin of grapes and blueberries, reduced pro-inflammatory IL-6 and TNF-α production via inhibiting STAT1 and STAT3, as well as NF-κB pathways. Additionally, oral administration of Resveratrol suppressed microglial activity associated with the production of cortical amyloid plaques in a mouse model of cerebral amyloid deposition [45]. It is worth mentioning that Resveratrol has undergone a phase II clinical trial to investigate its beneficial role in delaying or altering the deterioration of memory and daily functioning in AD [85].

Activator protein-1 (AP-1) is also another transcription factor that regulates pro-inflammatory genes, including COX-2 and iNOS, and this signaling is inhibited by Sulforaphane, leading to reducing the expression of many inflammatory mediators and pro-inflammatory cytokines [47]. Indeed, multiple transcription factors are potential targets of herbal medicines as the mutations of transcription factors are one of the causes of neurodegenerative diseases, including AD.

##### Nuclear Receptors (NRs)

Nuclear Receptors are responsible for regulating microglia phenotypes by activating transcription factors such as Peroxisome proliferator-activated receptors (PPARs) and nuclear factor erythroid 2-related factor 2 (Nrf2) [86]. PPARs are a nuclear receptor family composed of three subtypes, one of which is PPARγ, which suppresses the expression of pro-inflammatory mediators such as TNF-α, IL-6, IL-1β, and IL-12 while also promoting the production of anti-inflammatory cytokines such as TGF-β and IL-10 [87]. PPARγ agonists, such as β-caryophyllene and Curcumin, have been shown in pre-clinical trials to alter microglia polarization to the M2 phenotype, as shown in Table 1. Moreoever, it is worth mentioning that Curcumin has been clinically studied. Phase II clinical trials were carried out, one for treating patients with mild to moderate Alzheimer’s disease [88] and the other for studying the combination of Curcumin and Ginkgo for treating mild to severe dementia [89]. The beneficial effects of PPARγ agonists are proposed to be due to the suppression of microglial pro-inflammatory activity as well as the promotion of their phagocytic activity [90,91].

In addition, Nrf2 is a nuclear receptor that governs antioxidant responses initiated in oxidative damage, which is a feature of many neurodegenerative disorders [92]. Nrf2 expression in macrophages directly suppresses inflammation by blocking RNA polymerase II to IL-6 and TNF, as well as modulating antioxidative defense proteins such as heme oxygenase-1 (HO-1) [93]. As a result, Nrf2 activation is hypothesized to be involved in neuroprotection for Alzheimer’s disease patients. An in-vitro study conducted by Yeon Seo, Ji et al. [52] showed that Andrographolide activates the Nrf2/Keap1- mediated HO-1 signaling pathway, leading to a decrease in the expression of iNOS and COX-2 in BV-2 cells [52].

##### Protein Kinases (PKs)

MAPKs are one of the most important kinase groups in inflammatory cells. They include Extracellular signal-regulated kinase (ERK_1/2_), also known as p44/42 MAPK, and c-Jun N-terminal kinase (JNK), as well as p38 MAPK pathways [94]. Activation of these MAPK pathways causes phosphorylation of nuclear transcription factors and other cytoplasmic protein kinases, which results in increased expression of target inflammatory genes. For example, p38 MAPK activation via multiple pathways is necessary for the productions of IL-1, IL-6, TNF-α, COX-2, and iNOS, implying that p38 MAPK activity is associated with the hallmark lesions of Alzheimer’s disease [94]. Hence, targeting these activations through suppressing phosphorylation of the proteins is a proposed mechanism of many herbal medicines, such as Curcumin and Aromatic-turmerone [38,41]. Furthermore, Silibinin, Triptolide, Xanthoceraside, and Eriodictyol are natural plants that have been studied in-vitro and in-vivo to treat AD by inhibiting different MAPK pathways, as summarized in Table 1.

Similarly, the mammalian target of rapamycin (mTOR) kinase, a member of the phosphatidylinositol 3-kinase-related kinase (PIKKs) protein kinase family, is implicated in the neuroinflammation process. mTOR activation will eventually result in the activation of the NF-κB signaling pathway. As a result, blocking mTOR can reduce microglial cell activation and enhance M2 phenotypic conversion. Paeoniflorin, a traditional Chinese herb, has been proven in a rat model to suppress the mTOR/NF-κB pro-inflammatory pathway [56].

##### Cytokines

Cytokines are small proteins that have a role in controlling innate and adaptive immune responses. They are also involved in cell growth, survival, differentiation, and activities regulation [95]. Various types of CNS cells, including tissue infiltrating immune cells, neurons, and astrocytes, have been identified as CNS cytokine sources. However, microglia appears to be a major source of both pro-inflammatory and immune-regulatory cytokines. Several cytokines and their receptors have been discovered to exist and function in the CNS. TNF-α, IFNs, ILs including IL-1, -2, -3, -4, -6, -10, -12, -15, and -18, TGFβ, and CSFs are some of them [96]. During CNS inflammation, microglia produce two main pro-inflammatory cytokines, IL-1 and TNF-α, which are involved in BBB disruption [97]. Thereby, inhibiting activation of microglia and attenuating production of pro-inflammatory and anti-inflammatory cytokines are proposed mechanisms of many phytochemical compounds to treat AD, as shown in Table 1. For example, Oridonin extracted from *Rabdosia rubescens* has been shown to reduce NO production as well as the attenuation of iNOS, IL-1β, and IL-6 expressions that are involved in the development of neuroinflammation and neurodegeneration [59]. Moreover, Luo et al. (2018) found that the administration of Paeoniflorin, derived from *Paeonia lactiflora*, inhibits the productions of IL-1β, IL-6, TNF-α, and NO, while upregulating IL-10 and TGF-β1, which promote the transition of M1 to M2 phenotypes in microglia [56].

#### 3.1.2. PD

**Figure 3:**
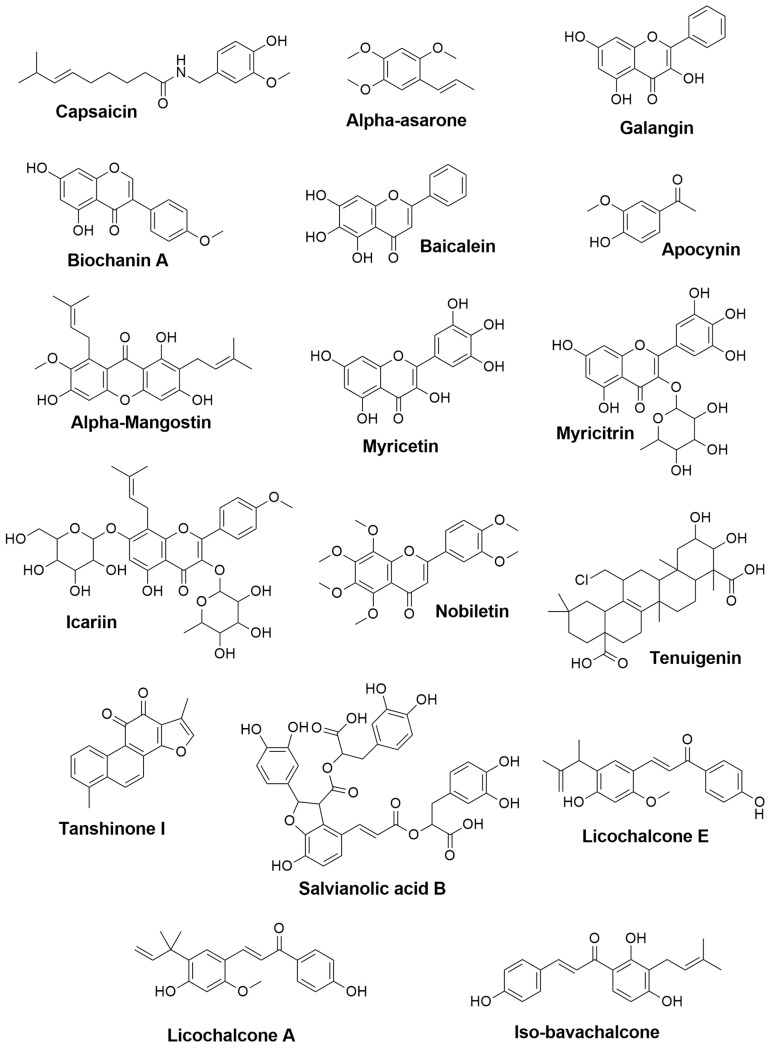

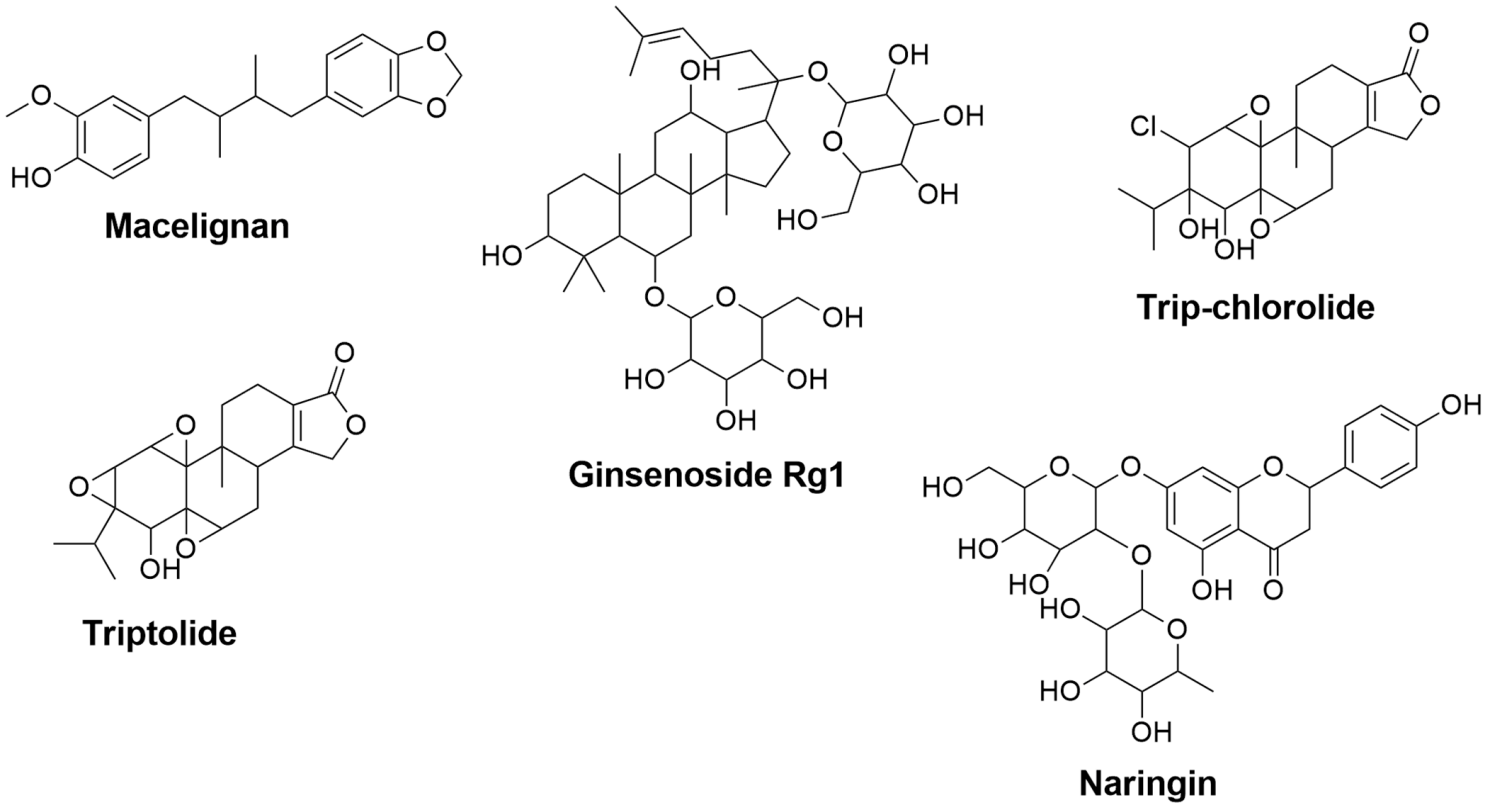
The 2D chemical structures of the neuroprotective phytochemical for PD treatments.

Parkinson’s disease (PD) is a progressive age-related neurodegenerative condition characterized by resting tremors, muscle rigidity, bradykinesia, and postural reflex deficits [98]. There is scientific proof that oxidative stress, peptide misfolding, and the death of dopaminergic neurons in the substantia nigra pars compacta are the fundamental features of Parkinson’s disease pathophysiology [99]. Although Levodopa is the gold standard for symptomatic management of Parkinson’s disease, long-term usage has been linked to the development of dyskinesia. Besides that, there are no pharmacological options that provide neuroprotection or slow the onset of PD. As a result, more efforts are required to discover therapy methods that alter the course of PD progression as well as relieve symptoms [100]. Therefore, numerous studies on phytochemical compounds have been conducted to investigate secondary metabolites’ efficacy and mechanisms in treating PD, some of which will be summarized in Figure 3 and addressed below.

##### Pattern Recognition Receptors (PRRs)

Rui W et al. (2020) [101] demonstrated that Baicalein, a flavonoid extracted from *Scutellaria baicalensis* Georgi, could reverse MPTP-induced motor dysfunction and dopaminergic neurons loss in mice model via blocking the NLRP3/caspase-1/gasdermin D pathway, which suppresses the disease-associated pro-inflammatory cytokine [101]. Moreover, Tenuigenin showed increased striatal dopaminergic levels and reduced motor impairment in the MPTP-induced mice model by suppressing NLRP3 inflammasome activation and decreasing caspase-1 and IL-1β productions as summarized in Table 2 [102].

##### Transcription Factors (TFs)

Kim et al. (2015) [111] revealed that prophylactic therapy with α-asarone inhibits microglial activation by blocking the NF-κB pathway, which improves PD-like behavioral impairment [106]. Likewise, several phytochemical compounds are have been reported to treat PD in pre-clinical experiments via targeting the transcription factor, NF-κB, such as Apocynin, α-Mangostin, Myricetin, Icariin, Nobiletin, Isobavachalcone, and Ginsenoside Rg1, among other herbs, as shown in Table 2. Further, STAT1 is a potential target for Parkinson’s disease therapy; Apocynin, a herb derived from *Picrorhiza kurroa*, has been shown to alleviate learning and memory impairments in the mice model through suppression of STAT1 and NF-κB signaling pathways [111].

##### Nuclear Receptors (NRs)

In PD patients, clinical trials with pioglitazone, a PPARγ agonist, have shown encouraging results [131]. Moreover, Macelignan is a plant-derived from *Myristica fragrans* that exhibits a PPARγ agonist activity and has been demonstrated to protect dopaminergic neurons [124]. Nrf2, a nuclear receptor that defends against oxidative stress and inflammatory process, is a target for Licochalcone E herb extracted from *Glycyrrhiza inflata*. Lico-E activates the Nrf2-antioxidant response element (ARE) system and up-regulates HO-1 [132].

##### Protein Kinases (PKs)

Kim et al. (2019) [121] found that Galangin suppressed the phosphorylation of p38 MAPK and JNK pathways, which significantly reduced the production of NO, iNOS, and IL-1β [107]. Similarly, phytochemical compounds such as Biochanin A, Baicalein, Myricetin, Macelignan, and Ginsenoside Rg1, which are listed in Table 2, have also been shown in pre-clinical studies to treat PD via targeting MAPKs pathways. Further, suppressing the phosphorylation of ERK_1/2_ is one of the mechanisms of Licochalcone A, according to in-vitro and in-vivo experiments in which the LPS-stimulated production of pro-inflammatory mediators and microglial activation was inhibited [121].

##### Cytokines

Growing evidence revealed that activation of microglia in the PD brain resulted in higher expression of pro-inflammatory cytokines, in which the productions of IL-1β, IL-6, and TNF-α were enhanced in activated microglia [133]. Several phytochemical compounds have been studied pre-clinically to treat PD, as shown in Table 2, and it has been noted that they exert their activity by inhibiting pro-inflammatory cytokines releases, such as Capsaicin and Icariin.

#### 3.1.3. MS

Multiple Sclerosis (MS) is a chronic degenerative neuroinflammatory disease that affects the central nervous system (CNS) and manifests in a range of clinical presentations. It is characterized by immunological abnormalities that result in myelin degradation in grey and white matter plaques [134,135]. The neurological symptoms are associated with the visible inflammatory lesions made up of lesser amounts of microglia and other types of cells that are all involved in the demyelinating process.

**Figure 4:**
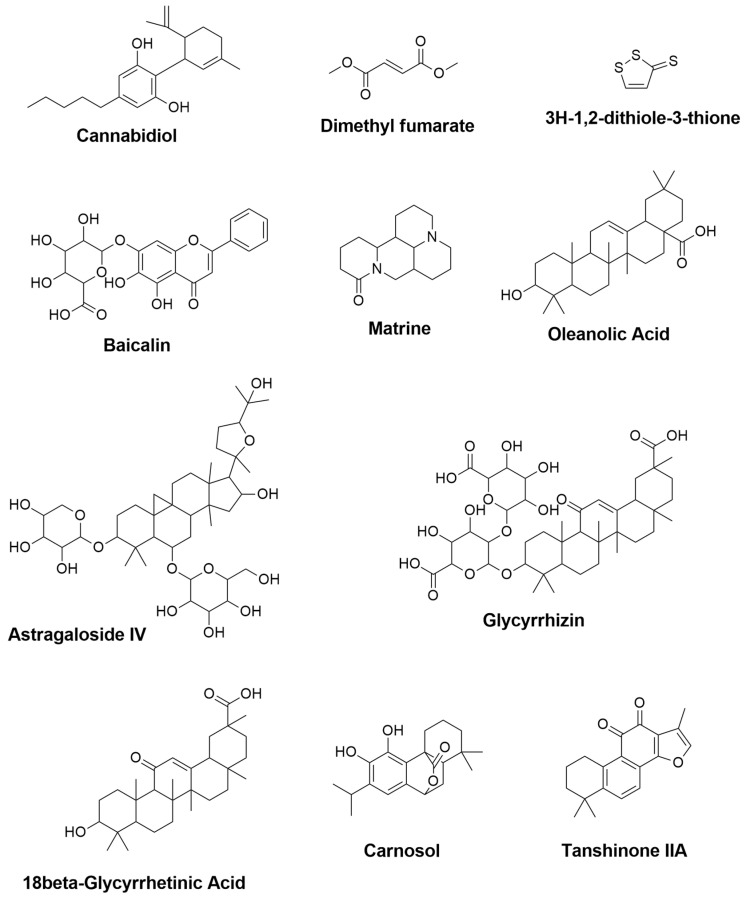
The 2D chemical structures of the neuroprotective phytochemicals used for MS treatments.

Currently, there is no cure for MS; however, there are two available approaches for management. The first is known as disease-modifying drugs, which include recombinant interferon β-1a and β-1b (e.g., Avonex and Betaferon), in addition to glatiramer acetate [136]. These agents are used to prevent relapses and improve neuropsychological deficits by inhibiting gamma interferon and enhancing the production of anti-inflammatory cells [137,138]. The second approach involves utilizing γ-aminobutyric acid type B (GABA-B) receptor agonists (e.g., baclofen) and α2 adrenergic receptor agonists (e.g., tizanidine) to manage MS symptoms such as pain and spasticity, with moderate benefits [139,140]. Multiple research, on the other hand, has studied the role of bioactive metabolites (Figure 4) as a therapeutic alternative for MS, which will be mentioned below.

##### Pattern Recognition Receptors (PRRs)

According to Peng H et al. (2016) [141], Dimethyl fumarate, the methyl ester of fumaric acid, is strongly suppressed NF-κB activation, besides other pathways, leading to a reduction of pro-inflammatory cytokines and chemokines production, which eventually improves the survival of oligodendrocytes and neurons [141]. It is worth mentioning that Dimethyl fumarate has been approved by the FDA to manage relapsing-remitting MS.

##### Nuclear Receptors (NRs)

Some natural plants have been studied to treat MS through activating Nrf2, which modulates the anti-oxidant stress response. As an example, Dimethyl fumarate, it has been reported that activation of Nrf2 receptor will lead to inhibit the phosphorylation of NF-κB signaling [142]. Moreover, Foresti et al. (2013) [143] identified Carnosol, a traditional medicine derived from Rosmarinus officinalis [Rosemary] and Salvia officinalis, to be a potent activator of the Nrf/Ho-1 pathway [143].

##### Protein Kinases (PKs)

18β-Glycyrrhe acid derived from Glycyrrhiza glabra is demonstrated by Zhou J. et al. (2015) [144] in a mice model to block the release of neurotoxic pro-inflammatory mediators induced by IFN-γ through inhibiting the phosphorylation of the MAPK pathways, ERK_1/2_ and p38 in microglia [144].

##### Cytokines

Most of the natural plants proposed to treat MS share the inhibition of IFN-γ cytokines, which function as effector cells damaging CNS cells by phagocytosis and the release of cytotoxic substances such as glutamate, nitric oxide, superoxide, and pro-inflammatory cytokines [145]. As shown in Table 3, Cannabidiol, 3H-1,2-dithiole-3-thione, Oleanolic Acid, Astragaloside IV, and Glycyrrhizin are all compounds that have been studied and found to suppress IFN-γ.

Glycyrrhizin, a compound extracted from licorice root, was studied by Sun Y. et al. (2018) [146] who showed that glycyrrhizin had an anti-inflammatory effect against MS through suppressing microglial M1 activation via reducing TGF-β1, IFN-γ, TNF-α, IL-17A, and IL-6 cytokines while increasing IL-4 [146]. On the other hand, Sativex® [Nabiximols®], a derived mixture of delta-9-tetrahydrocannabinol and Cannabidiol, is an investigational product in Phase III for the spasticity and pain associated with MS in the US [147].

**Table 3:** Modulatory Mechanisms of the Neuroprotective Phytochemicals used to Treat MS Based on in-silico Predictions and in-vitro and in-vivo Reported Studies.

Compound Names	Compound Natural Sources	In-Silico Anti-inflammatory Prediction		Modulatory Mechanism of Microglia Polarization	
		Pa	Pi	In-Vitro	In-Vivo
Cannabidiol	*Cannabis sativa*	0.427	0.082	-	Reduction of TNF- α, IFN-γ and IL-17 [148]
Dimethyl fumarate	*Fumaria officinalis*	0.469	0.066	Upregulation of gene expression for IGF-1 and MRC1 [149] Activation of Nrf2 and modulation of NF-κB pathways, leading to reduction of TNF- α and IL-12 productions [141]	-
3H-1,2-dithiole-3-thione	*Cruciferous* plants	0.945	0.004	Suppression of IFN-γ and IL-17 [150]	-
Baicalin	*Scutellaria baicalensis*	0.674	0.019	-	Reduction of IFN-γ, and elevation of IL-4 [151] Inhibition of STAT/NF-κB pathways [152]
Matrine	*Radix sophorae flavescentis*	NA	NA	-	Reduction of caspase-3, HSPB5 (alpha B-crystallin), and IL-1β [153]
Oleanolic Acid	*Olea europea, Aralia chinensis*, and *Rosa woodsia*	0.819	0.005	Suppression of TNF-α, COX-2, and iNOS [154]	Attenuation of TNF-α [154] Reduction of IFN-γ and TNF-α, and elevation of IL-10 [155]
Astragaloside IV	*Astragalus membranceus*	0.774	0.009	-	Downregulation of iNOS, IFN-γ, TNF-α and IL-6 [156]
Glycyrrhizin		0.849	0.005	-	Reduction of TNF-α, IFN-γ, IL-17A, IL-6 and TGF-β1 and elevation of IL-4 [146]
18β-Glycyrrhetinic Acid	*Glycyrrhiza glabra*	0.863	0.005	-	Suppression of MAPK signal pathway [144] Reduction of TNF- α and IL-1β [157]
Carnosol	*Rosmarinus officinalis* and *Salvia pachyphylla*	0.594	0.033	Reduction of NO and TNF-α levels [143]	Reduction of iNOS and elevation of ARG-1 [158]
Tanshinone IIA	*Salvia miltiorrhiza*	0.432	0.080	-	Downregulation of IL-17 and IL-23 [159]

NA: not applicable.

### 3.2. Target Prediction 

We have investigated the possible targets of the bioactive metabolites of 54 plants using a Molinspiration webserver that predict the probability of the compound’s activity as G protein-coupled receptors ligand, ion channel modulator, a kinase inhibitor, nuclear receptor ligand, protease inhibitor, and enzyme inhibitor.

#### 3.2.1. GPCR Ligand

G protein-coupled receptors (GPCRs) expressed by microglia had already been exhibited to regulate various aspects of their activation process, such as cell proliferation, migration, and differentiation into M1 or M2 phenotypes [160]. GPCRs, among these numerous different receptor types, play an important role in the modulation of different components of microglial activation. As a direct consequence, the involvement of GPCRs and their subtypes in neurological diseases has been implicated in many studies. Furthermore, many other unstudied GPCR subtypes are highlighted in microglial activation and need to be investigated for their potential therapeutic and molecular activity in Alzheimer’s disease [161,162]. Several types of research have concluded that GPCRs are novel targets for treating neuropsychiatric illnesses such as anxiety, depression, and cognition in Alzheimer’s disease, Parkinson’s disease, Huntington’s disease, and schizophrenia.

As shown in Table 4, only compounds Epigallocatechin-3-gallate, Andrographolide, Paeoniflorin, Oridonin, Dihydromyricetin, 4-O-methylhonokiol, Silibinin, Triptolide, Eriodictyol, Piper-longumine, Capsaicin, Tenuigenin, Iso-bavachalcone, Trip-chlorolide, Triptolide, Naringin, Cannabidiol, Matrine, Oleanolic Acid, 18β-Glycyrrhetinic Acid, and Carnosol were active at G protein-coupled receptors (GPCRs). Furthermore, compounds Andrographolide, Cannabidiol, and Carnosol were the most active compounds with scores of 0.32, 0.35, and 0.52, respectively.

Cannabinoid receptor 2 (CB2R) is a subfamily of GPCRs found on cell membranes. Although CB2R is abundant on peripheral immune cells, it is only found in very small amounts in the normal brain, primarily in microglia [163]. Interestingly, Cheng Z et al. (2014) [58] Founded that β-Caryophyllene intragastric administration (48 mg/kg, for 10 weeks) to APP/PS1 rats might prevent cognitive impairments and reverse neurodegeneration [58]. This was linked to a reduction in microglial M1 activation and inflammatory cytokines via the CB2R and PPAR- pathway [58]. However, in the Molinspiration biological predictions, our results showed that β-caryophyllene is not active as GPCR with a result of –0.34, as shown in Table 4. 

In-silico predictions suggested compounds Andrographolide, Cannabidiol, and Carnosol are active as GPCR-targeting. However, the reported studies have not investigated these possible targets suggesting further mechanistic studies are warranted. 

#### 3.2.2. Ion Channel Modulators

Microglial functions, including the proliferation, morphological alterations, migration, cytokine release, and reactive oxygen species generation, are all regulated by ion channels and transporters, which regulate ionic flux [164]. In microglial cells, ion channel expression is carefully controlled, with most ion channel types expressing differently depending on the cells’ functional state. Even though microglia are non-excitable cells, the abundance of voltage-gated ion channels shows that they play an important role in both normal and pathological conditions. Inflammation in the brain is a hallmark of Alzheimer’s disease, and multiple studies have shown that microglia can directly interact with neurons to cause inflammation [165].

As illustrated in Table 4, the findings of Resveratrol, Epigallocatechin-3-gallate, Andrographolide, Paeoniflorin, β-caryophyllene, Oridonin, Dihydromyricetin, Triptolide, Isobavachalcone, Tripchlorolide, Triptolide, Carnosol, and Tanshinone IIA suggest that these bioactive metabolites could modulate ion channels; however, inadequate published data is investigating phytochemical compounds as ion channel modulators.

As microglia ion channels are key regulators of microglial function and morphology. New evidence on the presence of specific ion channel localization on microglia and the possibility of enhanced ion channel expression in neurodegeneration may open up a new method for selectively targeting microglia and reducing the ongoing inflammatory process [166]. Among the six potential transient receptors (TRP) subfamilies, only the TRPC (canonical), TRPV (vanilloid), TRPM (melastatin) are expressed in microglia [167]. Capsaicin, a TRPV1 agonist, has been demonstrated by Young C et al. (2017) [105] to be useful in treating Parkinson’s disease. Using the in-vivo model, Capsaicin (0.5 mg/kg, i.p.) was found to restore nigrostriatal dopaminergic neurons in MPTP-injected mice, resulting in improved motor function. This, however, did not match our in-silico predictions as shown in Table 4 that Capsaicin had activity as Ion Channel Modulator with a score of −0.15 [105].

Despite the lack of studies that evaluate these natural products, the in-silico prediction illustrated that β-caryophyllene, Oridonin, and Tripchlorolide are considered ion channel modulators with the activity of 0.28, 0.27, and 0.24, respectively.

#### 3.2.3. Kinase Inhibitors

Kinases have become attractive drug targets because they are involved in nearly all cellular activities, such as cell growth, survival, proliferation, differentiation, and metabolism, and dysregulation of their activity has been linked to a variety of diseases, including CNS disorders such as AD, PD, and MS [168].

Unfortunately, most of the compounds showed no activity as a kinase inhibitor. However, Yang et al. (2017) [54] suggested that the Andrographolide suppressed NF-κB nuclear translocation by suppressing NF-κB phosphorylation in BV-2 cells, which were supported by our in-silico study [54]. Moreover, Leung et al. (2005) [169] studied the novel mechanism of inhibition of NF-κB DNA-binding activity by diterpenoids found in the compound Oridonin to treat inflammatory diseases [169]. However, the study did not find Oridonin to be active as a kinase inhibitor. Nevertheless, Oridonin works as a Nuclear Receptor Ligand and Enzyme Inhibitor based on Molinspiration biological predictions. Additionally, using the prediction analysis, only Epigallocatechin-3-gallate, Dihydromyricetin, Silibinin, Quercetin, Apigenin, Galangin, Baicalein, Myricetin, Myricitrin, and Nobiletin showed a good activity as kinase inhibitors. Moreover, Quercetin and Myricetin were the most active, with a score of 0.28 for both. Goldmann et al. demonstrate that 18β-Glycyrrhetinic Acid targeted the MAPK, but this did not represent our in-silico prediction [170].

#### 3.2.4. Nuclear Receptor Ligand

Nuclear receptors have attracted a lot of attention in the last 10 years as prospective therapeutic targets for neurodegenerative diseases. Effective treatments for progressive neurodegenerative disorders including Alzheimer’s disease, Parkinson’s disease, Huntington’s disease, and ALS have eluded researchers for years, making non-traditional therapeutic targets like nuclear receptors an appealing alternative. The involvement of nuclear receptors in several neurodegenerative disorders, most notably Alzheimer’s disease, has been studied extensively in mice models of disease and several therapeutic studies [86].

Our in-silico predictions suggest that Curcumin, Resveratrol, Pterostilbene, Epigallocatechin-3-gallate, Andrographolide, Paeoniflorin, β-caryophyllene, Oridonin, Dihydromyricetin, 4-O-methylhonokiol, Silibinin, Triptolide, Eriodictyol, Quercetin, Apigenin, Capsaicin, Galangin, Biochanin A, Baicalein, α-Mangostin, Myricetin, Myricitrin, Licochalcone E, Licochalcone A, Isobavachalcone, Triptolide, Naringin, Cannabidiol, Baicalin, Oleanolic Acid, 18β-Glycyrrhetinic Acid, Carnosol, and Tanshinone IIA were active as nuclear receptor ligand as summarized in Table 4.

Zun-jing et al. (2016) [86] reported that Curcumin inhibited the NF-κB signaling pathway and reduced the production of pro-inflammatory mediators from M1 microglia by specifically targeting PPAR-γ which is a Nuclear Receptor, and this was obvious in the Molinspiration biological predictions with an activity of 0.12 [86]. Moreover, Cheng et al. (2014) [40] showed that β-caryophyllene intragastric treatment (48 mg/kg, for 10 weeks) to APP/PS1 mice could prevent cognitive decline and reverse neurodegeneration through the activation of the CB2R and PPAR-pathways. This correlates with the reduction in microglial M1 activation and inflammatory cytokines [40]. Interestingly, all these results were supported by the Molinspiration webserver. Moreover, as shown in Table 4, some of the data were favorable as a Nuclear Receptor ligand, especially for compound PD-4. The results of the Galangin matched those of Min-ji and his colleagues in their 2017 study in which authors suggest in LPS-stimulated BV-2 cells, Galangin is a well-known PPAR activator that inhibits M1 inflammatory responses and increases the Nrf2/CREB signaling pathway from 10 to 50 μM [58]. Additionally, Sativex® (Sativex-like combination of Phytocannabinoids) therapy alone exhibited potential results in TMEV-IDD (Theiler’s murine encephalomyelitis virus-induced demyelinating disease) models as a modulatory drug for increasing microglia polarization to M2 phenotype to establish cytoprotective milieu. The therapeutic effects of Sativex may be due to (tetrahydrocannabinol-botanical drug substance) THC-induced upregulation of both CB1R and CB2R expression, as well as CBD-induced PPAR activation, and this matched the in-silico of Cannabidiol which showed a good activity (0.38) as nuclear receptor ligand [171]. Furthermore, compounds Andrographolide, Oridonin, Oleanolic Acid, 18β-Glycyrrhetinic Acid, and Carnosol demonstrated high scores of 0.94, 0.73, 0.77, 0.79, and 0.51 as nuclear receptor ligand, respectively. 

#### 3.2.5. Protease Inhibitors

Gene transcription, the initiation process of precursor forms, and interactions with endogenous protease inhibitors are all mechanisms that closely regulate protease activity. Once activated, proteases can cause irreversible breakage of peptide bonds in various proteins. Some substrates are inactivated after cleavage, while others are activated to gain new functionalities. As a result, microglial proteases are thought to have both positive and negative effects. According to Table 4, only compounds Epigallocatechin-3-gallate, Andrographolide, Paeoniflorin, Oridonin, Dihydromyricetin, Silibinin, Triptolide, Tenuigenin, Isobavachalcone, Tripchlorolide, Triptolide, Naringin, Matrine, Oleanolic Acid, and Glycyrrhizin appear to have good activity as protease inhibitors. Defects in proteostasis are thought to be associated with various neurodegenerative disorders, including Parkinson’s disease. While the proteasome fails to destroy large protein aggregates, such as alpha-synuclein (α-SYN) in PD, drug-induced autophagy can effectively remove clusters and prevent dopaminergic neuron degeneration. As a result, maintaining these pathways is critical for preserving all cellular functions that rely on a properly folded proteome [172]. The Molinspiration analysis indicated that Tenuigenin, Isobavachalcone, Tripchlorolide, Triptolide, and Naringin act as Protease Inhibitors.

#### 3.2.6. Enzyme Inhibitors

The aggregation of misfolded amyloid-β and hyperphosphorylated tau and α-synuclein are linked to the pathogenesis of AD and PD, respectively. To cure the diseases, multiple small molecules have been developed to regulate the aggregation pathways of these amyloid proteins. In addition to controlling the aggregation of amyloidogenic proteins, maintaining the levels of the proteins in the brain by amyloid degrading enzymes (ADE); neprilysin (NEP), insulin-degrading enzyme (IDE), asparagine endopeptidase (AEP), and ADAM10 is also essential to cure AD and PD. Therefore, numerous biological molecules and chemical agents have been investigated as either inducers or inhibitors against the levels and activities of amyloid degrading enzymes [173]. All the AD and PD compounds showed enzyme inhibitor activity except Aromatic-turmerone, Xanthoceraside, Esculentoside A. α-asarone, Apocynin, Icariin, Tanshinone I, Salvianolic acid B, Licochalcone E, and Ginsenoside Rg1. 

Moreover, reactive oxygen species (ROS) possess a physiological role in various cellular regulation processes. Antioxidant enzyme therapy may be advantageous for treating MS as ROS scavengers may interfere at numerous levels during the formation of MS lesions [174]. Cannabidiol, Baicalin, Matrine, Oleanolic Acid, 18β-Glycyrrhetinic Acid, Carnosol, and Tanshinone IIA demonstrated activity as enzyme inhibitors with an activity of 0.33, 0.26, 0.06, 0.65, 0.70, 0.37, and 0.08, respectively, as shown in Table 4.

### 3.3. Absorption, Distribution, Metabolism, and Excretion (ADME)

ADME properties were predicted using SwissADME, an online web server. Furthermore, the BBB can prevent chemicals from entering the brain and acts as a natural barrier against numerous poisons and infected cells in the bloodstream, but it also restricts the uptake of diagnostic and therapeutic substances in the brain, diminishing therapeutic efficiency and targeted delivery, therefore, small (often less than 500 Da) and lipophilic compounds can effectively penetrate the BBB and enter the brain. Thus, as disease-targeting strategies molecular weight (MW), blood-brain barrier penetration (BBB), high solubility (logS), and P-glycoprotein substrate, all are essential characteristics of the drug to be promising as a neuroprotective molecule [175].

#### 3.3.1. Molecular Weight (MW)

Considering Lipinski’s rule limit of MW of 500 g/mol, all compounds were within the recommended range, which improves their chances to be absorbed orally in the gastrointestinal tract except for Hesperidin, Xanthoceraside, Esculentoside A, Icariin, Tenuigenin, Salvianolic acid B, Ginsenoside Rg1, Naringin, Astragaloside IV, and Glycyrrhizin, which have molecular weights of 610.56, 1141.29, 973.11, 676.66, 537.13, 718.61, 801.01, 580.53, 784.97, and 822.93 g/mol, respectively [176].

#### 3.3.2. Blood-Brain Barrier (BBB) Permeability

All the studied compounds could not cross the blood-brain barrier (BBB) except for Aromatic-turmerone, Resveratrol, Pterostilbene, 4-O-methylhonokiol, Piperlongumine, Capsaicin, α-asarone, Apocynin, Tanshinone I, Licochalcone E, Licochalcone A, Macelignan, Cannabidiol, Matrine, Carnosol, and Tanshinone IIA. Moreover, these sixteen compounds possess an advantage of blood-brain barrier penetration that allows them to be used in treating neurodegenerative diseases and targeting microglia [177]. Furthermore, α-asarone is one of the most studied compounds to cross the blood-brain barrier in more than one scientific study as an effective treatment for Parkinson’s disease. For example, according to Chinese medicine, Xiao et al. (2015) [178] showed that α-asarone had been used to treat dementia, amnesia, and stroke as an orifice-opening medicinal because of the adequate and appropriate BBB permeability [178]. Similarly, Carnosol can cross through the BBB and subsequently produce an anti-inflammatory effect on M1 microglia in the CNS, according to Xing Li et al. (2018). [158]

#### 3.3.3. Solubility (Log S)

The aqueous solubility of substances that have a direct impact on oral absorption is referred to as Log S. Within the specified range (−6.5 to 0.5), all compounds demonstrated soluble to moderate solubility except for Nobiletin, Tanshinone I, Astragaloside IV, and Tanshinone IIA with log S values of −6.82, −6.91, and −6.71 which were poorly soluble.

#### 3.3.4. P-glycoprotein Substrate

P-glycoprotein (P-gp) has emerged as the transporter that poses the largest barrier to innovative neuroprotective drug delivery among the BBB’s reported transporters. All the compounds are not a P-glycoprotein substrate except for Andrographolide, Paeoniflorin, Oridonin, Hesperidin, Triptolide, Eriodictyol, Xanthoceraside Esculentoside A, Icariin, Tenuigenin, Ginsenoside Rg1, Tripchlorolide, Triptolide, Naringin, Astragaloside IV, Glycyrrhizin, 18β-Glycyrrhetinic Acid, Carnosol, and Tanshinone IIA. All ADME results are summarized in Table 5.

### 3.4. Toxicity and Safety Prediction for Neuroprotective Phytochemicals

#### 3.4.1. Inhibition of the Cytochromes P450

Herbs can accelerate or decrease the expected activity of prescribed medication, resulting in undesired side effects or therapeutic failure. Herbal active components can dramatically affect a drug’s pharmacokinetic and pharmacodynamic properties, raising concerns regarding herb-drug interactions. The inhibition or induction of cytochrome P450 (CYP450) has been proposed as one of the key mechanisms for herb-drug interactions. Thus, to evaluate the potential interactions between the bioactive metabolites of natural herbs and cytochrome P450 enzymes SwissADME webserver was utilized [179].

As shown below in Table 6, 4-O-methylhonokiol and Tanshinone IIA strongly inhibited all the CYP groups. Moreover, the safest compound that did not show any inhibition of cytochrome P450 was Aromatic turmerone, Sulforaphane, Epigallocatechin-3-gallate, Andrographolide, Paeoniflorin, Oridonin, Dihydromyricetin, Hesperidin, Triptolide, Xanthoceraside, Piperlongumine, and Esculentoside A, for the PD, they were Apocynin, Myricitrin, Icariin, Tenuigenin, Salvianolic acid B, Ginsenoside Rg1, Tripchlorolide, Triptolide, and Naringin moving to MS they were Dimethyl fumarate, 3H-1,2-dithiole-3-thione, Matrine, Oleanolic Acid, Astragaloside IV, Glycyrrhizin, and 18β-Glycyrrhetinic Acid.

#### 3.4.2. Organ Toxicity

During the development of new medicine, the most important consideration is always safety, which includes a variety of toxicities and adverse drug effects that should be assessed during the preclinical and clinical trial phases. Herein, we investigated the direct organ toxicity of bioactive metabolites using computational approaches [180].

We investigated the safety profile of all compounds by conducting toxicity prediction tests with the ProTox-II online tool. This server classified compounds into six toxicity classes [1,2,3,4,5,6], with class 1 being the most toxic and fatal, with an estimated lethal dosage (LD_50_) of 5, and class 6 demonstrating an LD_50_ > 5000, indicating the compound is non-toxic. All compounds’ LD_50_, organ toxicity (hepatotoxicity], toxicity endpoints [carcinogenicity, mutagenicity, immunotoxicity), were predicted, except compound Glycyrrhizin and 18β-Glycyrrhetinic Acid, which were inactive. Furthermore, the toxicity class and the estimated probability of each compound were provided. The oral toxicity prediction findings revealed that the safest compounds were Hesperidin, Apocynin, Tenuigenin, and Astragaloside IV, which were in class 6, and the majority of the compounds were in class 4 and 5, except for compounds Oridonin, and Quercetin, Myricetin, Dimethyl fumarate, and Matrine, which were in class 3. For the most toxic and fatal compounds, they were only compounds Triptolide and Capsaicin, Salvianolic acid B, Tripchlorolide, and Triptolide which were classified as 1 and 2. On the ProTox-II server, the majority of the compounds in Table 7. were predicted to be potentially immunogenic except for Aromatic-turmerone, Resveratrol, Sulforaphane, Epigallocatechin-3-gallate, Paeoniflorin, Dihydromyricetin, Eriodictyol, and Apigenin, Galangin, Biochanin A, Baicalein, Apocynin, Myricetin, and Tanshinone I, Dimethyl fumarate, 3H-1,2-dithiole-3-thione, Baicalin, Matrine, and Oleanolic Acid. Among the compounds investigated, 14 out of the 54 compounds were predicted to be carcinogenic, including Dihydromyricetin, Triptolide, Eriodictyol, Apigenin, Capsaicin, α-asarone, Baicalein, Myricetin, Myricitrin, Enuigenin, Tripchlorolide, Triptolide, Baicalin, Oleanolic Acid, and 18β-Glycyrrhetinic Acid. Furthermore, all compounds showed mutagenicity with probability values ranging from 0.51 to 0.99 except Dihydromyricetin, Apigenin, Capsaicin, Baicalein, Myricetin, Salvianolic acid B, and Baicalin. Finally, there was no remarkable hepatotoxicity except for Licochalcone E and Oleanolic Acid. To conclude, compounds Aromatic-turmerone, Resveratrol, Sulforaphane, Epigallocatechin-3-gallate, Paeoniflorin, Galangin, Biochanin A, Apocynin, Tanshinone I, Dimethyl fumarate, 3H-1,2-dithiole-3-thione, and Matrine could be considered safe according to ProTox-II online tool.

## 4. Materials and Methods

### 4.1. Literature Search

A systematic search was conducted in databases such as PubMed, Google Scholar, and Science Direct to identify relevant studies using key-words such as Microglia, Neurodegenerative diseases, Alzheimer disease, Parkinson disease, Multiple sclerosis, M1, and M2, Neuroprotective, ADME, in-vitro, in-vivo, in-silico, clinical trial. The reported phytochemicals in the studies that demonstrated neuroprotective effects via microglia modulation in neurodegenerative diseases (AD, PD, and MS) were selected.

### 4.2. Computational Analysis 

The 2D chemical structure of each bioactive constituent was drawn using Chemdraw, and the simplified molecular-input line-entry system (SMILES), was utilized to conduct the computational analysis. The following computational tools were used: PASS online, Molinspiration, SwissADME, and ProTox-II webservers.

#### 4.2.1. PASS Online 

The activity is predicted by finding similarities between the new compound chemical structure and a well-known biological active substrate in the database. The activity spectrum estimation algorithm uses a Bayesian method. The PASS prediction tool will predict the probability of active [Pa] to probability of inactive [Pi] ratio. According to leave-one-out cross-validation [LOO CV] estimation, the average prediction accuracy is around 95%. PASS prediction accuracy depends on detailed information on the biological activity spectrum for each molecule in the PASS training set, so the biological activity estimate is more accurate. The website ( www.way2drug.com, accessed on 25 May 2021) [181] can be accessed directly with the search term "PASS prediction" in multiple web browsers.

#### 4.2.2. Molinspiration 

Molinspiration (www.molinspiration.com, accessed on 26 December 2021) [182] is a free online tool that aids the internet chemistry community by calculating essential chemical characteristics and predicting bioactivity scores for the most important drug targets [GPCR ligands, kinase inhibitors, ion channel modulators, nuclear receptors]. A molecule with a bioactivity score greater than 0.00 is most likely to have significant biological activities, whereas values and scores less than −0.50 are considered inactive.

#### 4.2.3. SwissADME 

To enhance drug discovery, this webserver (www.swissadme.ch, accessed on 8 November 2021) [183] allows for computing physicochemical descriptors and estimating absorption, distribution, metabolism, and excretion [ADME] parameters, pharmacokinetic properties, druglike nature, and medicinal chemistry properties of one or more small molecules.

#### 4.2.4. ProTox-II

ProTox-II (http://tox.charite.de/protox_II, accessed on 8 November 2021) [184] uses a total of 33 models based on molecular similarity, fragment propensities, most frequent features, and [fragment similarity-based CLUSTER cross-validation] machine learning to predict various toxicity endpoints like acute toxicity, hepatotoxicity, cytotoxicity, carcinogenicity, mutagenicity, immunotoxicity. Toxicity classifications are determined using the globally harmonized system of classification of labeling of chemicals (GHS); toxic doses are frequently expressed as LD_50_ values in milligrams per kilogram of body weight. The median lethal dose (LD_50_) is the dose at which 50% of test subjects die after being exposed to a substance. The following are the classification and the (mg/kg) LD_50_ values.
Class 1:Fatal if swallowed [LD_50_ ≤ 5]Class 2:Fatal if swallowed [5 < LD_50_ ≤ 50]Class 3:Toxic if swallowed [50 < LD_50_ ≤ 300]Class 4:Harmful if swallowed [300 < LD_50_ ≤ 2000]Class 5:It may be harmful if swallowed [2000 < LD_50_ ≤ 5000]Class 6:Non-toxic [LD_50_ > 5000]

## 5. Conclusions and Future Directions

The reported biological activity of neuroprotective medicinal plants could result from the overall effects of several bioactive molecules on multiple targets that make it difficult to identify the specific biological activity of a phytochemical. Thus, in this study, we screened 54 phytochemicals that have been reported in-vitro and in-vivo to be neuroprotective against NDs, and several parameters important for drug design and development were evaluated.

One of the most crucial factors that limit the therapeutic applications of these phytochemicals for the treatment of NDs is the physicochemical properties. Thus, we have selected phytochemicals that exhibited a good pharmaceutical profile with 0 violation of the rule of five [ROF], and only 34 phytochemicals were selected. The second important criteria that were considered is the safety and toxicity profile; thus, phytochemicals classified as class 4 and above were chosen, and the selection included 27 phytochemicals that passed this criterion. Furthermore, since herb-drug interactions are as important as toxicity, we selected phytochemicals that exhibited no CYP enzymes inhibition, and phytochemicals are Aromatic-turmerone, Sulforaphane, Andrographolide, Piperlongumine, Apocynin, and 3H-1,2-dithiole-3-thione.

To conclude, natural products hold considerable promise for treating various NDs, even though numerous questions concerning their efficacy and safety remain unevaluated. After the screening of 54 phytochemicals with neuroprotective effects in microglia, we can draw a solid conclusion that Aromatic-turmerone, Sulforaphane, Andrographolide, Piperlongumine, Apocynin, and 3H-1,2-dithiole-3-thione are the most promising compounds that could be considered when designing novel biologically active anti-inflammatory agents to treat neurodegenerative diseases via targeting microglial polarization. These six compounds demonstrated excellent ADME properties, safety profile, and promising anti-inflammatory activity that could be utilized as lead compounds for further drug optimization and development.

## Figures and Tables

**Table 1:** Modulatory Mechanisms of the Neuroprotective Phytochemicals used to Treat AD Based on in-silico Computational Predictions and Reported in-vitro and in-vivo Studies.

Compound Names	Compound Natural Source	In-Silico Anti-inflammatory Prediction		Modulatory Mechanism of Microglia Polarization	
		Pa	Pi	In-Vitro	In-Vivo
Curcumin	*Curcuma longa*	0.677	0.019	Suppression of ERK_1/2_ and p38 MAPK pathways, and inhibition of IL-1β, IL-6, and TNF-α [38] Induction of HO-1 leading to Inhibition of NO, PGE_2_, and TNF-α [39] Activation of PPARγ pathway and inhibition of the NF-κB signaling pathway [40]	Activation of PPARγ pathway and inhibition of the NF-κB signaling pathway [40]
Aromatic-turmerone	*Curcuma longa*	0.584	0.035	Inhibition of the NF-κB, JNK, and p38 MAPK signaling pathways [41] Suppression of iNOS, COX-2, NO, PGE_2_, and NF-κB, besides attenuation the levels of TNF-α, IL-1β, IL-,6, and monocyte chemoattractant protein-1(MCP-1) [42]	Reduction of TNF-α and IL-1β [43]
Resveratrol	the skin of grapes and blueberries	0.554	0.042	Reduction of the expression of mPGES-1, a key enzyme in the synthesis of PGE_2_ [44]	Inhibition of the NF-κB, STAT1, and STAT3 pathways and inhibition of TNF-α and IL-6 secretions [45]
Pterostilbene	*Pterocarpus marsupium*, blueberries	0.508	0.054	Inhibition of the NLR family pyrin domain containing-3 (NLRP3)/caspase-1 inflammasome pathway, and reduction of TNF,-α, IL-6, and IL-1β [36]	Inhibition of NO, TNF-α, and IL-6 [46]
Sulforaphane	Cruciferous vegetables (e.g., cabbage mustard radish, and broccoli)	NA	NA	Inhibition of JNK/AP-1/NF-κB pathway and activation of Nrf2/HO-1 pathway [47]	Reduction of IL-1β and TNF-α [48]
Epigallocatechin-3-gallate	*Camellia sinensis*	0.623	0.027	Suppression of iNOS and NO [49] Suppression of TNFα, IL-1β, IL-6 and iNOS [50]	Inhibition of iNOS and COX-2 [51]
Andrographolide	*Andrographis paniculate*	0.845	0.005	Activation of Nrf2/Keap1-mediated HO-1 signaling pathway, and downregulation of NF-κB signaling pathway [52] Inhibition of PGE_2_ and TNF-α, and downregulation of iNOS and COX-2 [53] Inhibition of NF-κB signaling pathway and JNK-MAPK pathway [54]	-
Paeoniflorin	*Paeonia lactiflora*	0.578	0.036	Suppression of TNF-α, IL-1β, and IL-6. Inhibition of NF-κB signal activation [55]	Inhibition of IL-1β, IL-6, TNF-α, and NO. Upregulation of IL-10 and TGF-β1. Inhibition of mTOR/NF-κB signaling pathway, and activation of phosphatidylinositol-3-Kinase and Protein/Kinase B (PI3K/Akt) signaling pathway [56]
β-caryophyllene	*Myristica fragrans*, *Piper Nigrum*, *Ribes nigrum,* and *Syzygium aromaticum*	0.745	0.011	Upregulation of IL-10 and Arg-1, and reduction of L-1β, TNF-α, PGE2, iNOS and NO; Activation of the PPAR-γ pathway [57]	Activation of cannabinoid receptor 2 (CB2R) and PPARγ receptor [58]
Oridonin	*Rabdosia rubescens*	0.681	0.018	Reduction of NO and attenuation of expression of iNOS, IL-1β, and IL-6 [59]	Inhibition of NF-κB pathway [60]
Dihydromyricetin	*Ampelopsis*, *Pinus*, and *Cedrus* species	0.737	0.012	Inhibition of TLR4/NF-κB signaling pathway [61]	Activation of Adenosine monophosphate-activated protein kinase (AMPK)/NAD-dependent deacetylase sirtuin-1 [SIRT1] pathway [62] Inhibition of NLRP3 inflammasome [63]
4-O-methylhonokiol	*Officinalis icinalis*	0.446	0.074	Inhibition of NF-κB pathways [64]	Inhibition of NF-κB pathways [64]
Silibinin	*Silybum marianum*	0.667	0.020	-	Inhibition of MAPKs pathway [65]
Hesperidin	The peel of citrus fruits	0.691	0.017	Reduction of iNOS and NO [66] Reduction of NO, iNOS, TNF-α and IL-1β [67]	Inhibition of protein kinase B/glycogen synthase kinase-3β (AKT/GSK-3β) and attenuation of iNOS, NF-κB, TNF-α, IL-1β, IL-4, IL-6, and COX-2 [68]
Triptolide	*Tripterygium wilfordii*	0.698	0.016	Inhibition of TNF-α and IL-1β [69]	Suppression of MAPKs including p3,8, ERK_1/2_, and JNK [70]
Eriodictyol	A variety of fruits and herbs	0.691	0.017	Suppression of NF-κB [35]	Inhibition of TLR4, MAPKs, and PI3K/Akt, and activation of SIRT1; thus, blocking NF-κB pathway [35]
Xanthoceraside	*Xanthoceras sorbifolia*	0.753	0.010	Suppression of IL-1β and TNF-α through inhibition of NF-κB and MAPK pathways [71]	Suppression of MAPK and NF-κB pathways [72]
Piperlongumine	*Piper longum*	0.435	0.079	Inhibition of NF-κB pathway [73,74]	Inhibition of NF-κB pathway [72]
Esculentoside A	*Phytolacca esculenta*	0.857	0.005	Inhibition of NF-κB, MAPKs, and NLRP3 pathways [37]	Reduction of iNOS, COX-2, and TNF-α through inhibition of MAPKs pathway [75]
Quercetin	Fruits and vegetables (e.g., onions and apples)	0.689	0.017	Reduction of NO through inhibiting NF-κB pathway [76]	-
Apigenin	A variety of fruits and vegetables (e.g., *chamomile*, *tea*, and *oranges*)	0.644	0.024	Suppression of IFN-γ [77]	-

**Table 2:** Modulatory Mechanisms of Phytochemicals used to Treat PD Based on in-silico Computational Predictions and Reported in-vitro and in-vivo Studies.

Compound Names	Compound Natural Sources	In-Silico Anti-inflammatory Prediction		Modulatory Mechanism of Microglia Polarization	
		Pa	Pi	In-Vitro	In-Vivo
Capsaicin	*Capsicum*	0.266	0.196	-	Elevation of the expression of ciliary neurotrophic factor receptor alpha [CNTFRα] [103] Reduction of NO, iNOS, and IL-6 expressions, and elevation of Arg-1 and macrophage mannose receptor (CD206) [104] Reduction of TNF-α and IL-1β expressions [105]
α-asarone	*Acorus tatarinowii*	0.592	0.033	Inhibition of NF-κB [106]	Inhibition of NF-κB [106]
Galangin	*Alpinia officinarum*	0.689	0.017	Inhibition of MAPK and NF-κB signaling pathways [107] Inhibition of TNF-α, IL-6, IL-1β, and COX-2 through JNK and NF-κB pathways [108]	Inhibition of TNF-α, IL-6, IL-1β, and COX-2 through JNK and NF-κB pathways [108]
Biochanin A	Legume plants	0.588	0.034	Inhibition of TNF-α and IL-1β through MAPK pathway [109]	Inhibition of TNF-α and IL-1β through MAPK pathway [109]
Baicalein	*Scutellaria baicalensis Georgi*	0.674	0.019	Inhibition of TNF-α and IL-6 through MAPK and NF-κB signaling pathways [110]	Suppression of NLRP3/caspase-1/GSDMD pathway [101]
Apocynin	*Picrorhiza kurroa*	0.496	0.058	-	Inhibition of STAT1 and NF-κB pathways [111]
α-Mangostin	*Mangosteen pericarp*	0.694	0.017	Inhibition of NF-κB pathway [112]	Reduction of IL-6 and COX-2 [113]
Myricetin	*Turbinaria ornata*	0.720	0.013	Inhibition of MAPK and NF-κB signaling pathways [114]	Inhibition of MAPK and NF-κB signaling pathways [114]
Myricitrin	*Myrica cerifera*	0.762	0.009	-	Suppression of TNF-α [115]
Icariin	*Herba epimedii*	0.732	0.012	Reduction of TNF- α, IL-1β and NO through inhibition of NF-κB pathway [116]	Reduction of TNF- α, IL-1β and NO through inhibition of NF-κB pathway [116]
Nobiletin	Citrus fruits	0.694	0.017	Suppression of TNF-α, IL-1β and NO through inhibition of NF-κB pathway [117]	Attenuation of IL-1β production [118]
Tenuigenin	*Polygala tenuifolia*	0.841	0.005	Inhibition of NLRP3 inflammasome and downregulation of caspase-1, pro-IL-1β, and IL-1β [102]	Suppression of NLRP3 inflammasome [102]
Tanshinone I	*Radix salviae miltiorrhizae*	0.515	0.053	Suppression of TNF-α, IL-6, and IL-1β [119]	Attenuation of the increase of TNF-α, and reserving the increase of IL-10 [119]
Salvianolic acid B	*Salviae miltiorrhizae*	0.313	0.149	Reduction of TNF-α, IL-1β and NO productions [120]	Attenuation of the expressions of TNF-α, IL-1β, and NO [120]
Licochalcone E	*Glycyrrhiza inflata*	0.523	0.050	Activation of Nrf2/ARE-dependent pathway [107]	Activation of Nrf2/ARE-dependent pathway [107]
Licochalcone A	*Glycyrrhiza inflata*	0.740	0.011	Inhibition of ERK_1/2_ and NF-κB p65 through reduction of iNOS, COX-2, TNF-α, IL-1β, and IL-6 expressions [121]	Inhibition of ERK_1/2_ and NF-κB p65 through reduction of iNOS, COX-2, TNF-α, IL-1β, and IL-6 expressions [121]
Isobavachalcone	*Psoralea corylifolia*	0.778	0.008	Inhibition of NF-κB pathway through inhibition of TNF-α, IL-6, IL-1β, and IL-10 [122]	Reduction of IL-6 and IL-1β expressions [122]
Macelignan	*Myristica fragrans*	0.352	0.121	Suppression of MAPKs and NF-kB via the regulation of IkB [123]	Activation of PPAR-γ [124]
Ginsenoside Rg1	*Panax ginseng*	0.801	0.007	Inhibition of NF-κB and MAPK signaling pathways through attenuation of TNF-α, IL-1β, iNOS, and COX-2 mRNA and protein levels [125]	Inhibition of NF-κB and MAPK signaling pathways through reduction of TNF-α, IL-1β, and IL-6 [126]
Tripchlorolide	*Tripterygium wilfordii Hook F*	0.791	0.007	Attenuation of TNF-α, IL-1β, NO, iNOS, PGE_2_, and COX-2 [127]	-
Triptolide	*Tripterygium wilfordii Hook F*	0.698	0.016	Downregulation of NO, iNOS, TNF-α, and IL-1β [128]	-
Naringin	Grapefruit, Citrus fruits	0.700	0.016	-	Inhibition of IL-1β [129] Attenuation of TNF-α [130]

NA: not applicable.

**Table 4:** Target Predictions of the Neuroprotective Phytochemicals Used for AD, PD, and MS Treatments using Molinspiration Webserver.

Compound Names	Molinspiration						Reported Target
	GPCR ligand	Ion Channel Modulator	Kinase Inhibitor	Nuclear Receptor Ligand	Protease Inhibitor	Enzyme Inhibitor	
Curcumin	−0.06	−0.20	−0.26	0.12	−0.14	0.08	ERK1/2 and p38 MAPK IL-1β, IL-6, and TNF-α NO, PGE2 PPARγ, NF-κB
Aromatic-turmerone	−0.68	−0.46	−1.36	−0.14	−0.80	−0.25	NF−κB, JNK, and p38 MAPK iNOS, COX-2, NO, PGE2, NF-κB, TNF-α, IL-1β, IL-,6MCP-1
Resveratrol	−0.20	0.02	−0.20	0.01	−0.41	0.02	mPGES-1 NF-κB, STAT1, STAT3, TNF-α, IL-6
Pterostilbene	−0.13	−0.06	−0.12	0.08	−0.33	0.01	NLRP3, NO TNF,-α, IL-6, IL-1β
Sulforaphane	−0.35	−0.59	−1.98	−0.84	−0.72	0.44	JNK/AP-1/NF-κB Nrf2/HO-1, IL-1β, TNF-α
Epigallocatechin-3-gallate	0.16	0.02	0.06	0.33	0.13	0.25	iNOS and NO TNFα, IL-1β, IL-6, COX-2
Andrographolide	0.32	0.17	−0.01	0.94	0.26	0.81	Nrf2/Keap1-, NF-κB, TNF-α, iNOS, COX-2 JNK-MAPK
Paeoniflorin	0.24	0.16	−0.03	0.15	0.14	0.44	TNF-α, IL-1β, and IL-6, NF-κB TGF-β1, mTOR, PI3K/Akt
β-caryophyllene	−0.34	0.28	−0.78	0.13	−0.60	0.19	IL-10 and Arg-1, L-1β, TNF-α, PGE2. iNOS, NO CB2R, PPARγ
Oridonin	0.1	0.27	−0.19	0.73	0.08	0.53	NO, iNOS, IL-1β, IL-6
Dihydromyricetin	0.09	0.03	0.01	0.27	0.08	0.32	TLR4/NF-κB, AMPK, SIRT1, NLRP3
4-O-methylhonokiol	0.04	−0.00	−0.09	0.29	−0.23	0.06	NF-κB
Silibinin	0.07	−0.05	0.01	0.16	0.02	0.23	MAPKs
Hesperidin	−0.01	−0.59	−0.36	−0.20	−0.00	0.06	iNOS, NO, TNF-α, IL-1β AKT/GSK-3β iNOS, NF-κB, TNF-α, IL-1β, IL-4, IL-6, COX-2
Triptolide	0.11	0.09	−0.43	0.4	0.24	0.86	TNF-α, IL-1β, MAPKs p3,8, ERK1/2, and JNK
Eriodictyol	0.07	−0.20	−0.22	0.46	−0.09	0.21	TLR4, MAPKs, PI3K/Akt, SIRT1, NF-κB
Xanthoceraside	−3.77	−3.85	−3.90	−3.82	−3.74	−3.71	IL-1β and TNF-α, MAPK, NF-κB
Piperlongumine	0.21	−0.03	−0.07	−0.08	−0.05	0.08	NF-κB
Esculen-toside A	−3.50	−3.71	−3.73	−3.63	−3.16	−3.36	TNF-κB, MAPKs, NLRP3 iNOS, COX-2, TNF-α MAPKs
Quercetin	−0.06	−0.19	0.28	0.36	−0.25	0.28	NO, NF-κB
Apigenin	−0.07	−0.09	0.18	0.34	−0.25	0.26	IFN-γ
Capsaicin	0.03	−0.01	−0.28	0.01	−0.02	0.07	CNTFRα CD206 TNF-α and IL-1β
α-asarone	−0.71	−0.43	−0.72	−0.47	−0.97	−0.39	NF-κB IL (NADPH) oxidase-2 (NOX2)/NF-κB tyrosine kinase (SRC)/ERK PGE2, COX-2, NO, iNOS IL-6, IL-1β, and TNF-α
Galangin	−0.13	−0.21	0.19	0.28	−0.32	0.28	TNF-α and IL-1β
Biochanin A	−0.23	−0.59	−0.07	0.23	−0.66	0.07	TNF-α and IL-1β
Baicalein	−0.12	−0.18	0.19	0.17	−0.35	0.26	TNF-α and IL-6 NLRP3/caspase-1/GSDMD
Apocynin	−1.01	−0.54	−1.22	−1.04	−1.31	−0.59	STAT1 and NF-κB
α-Mangostin	−0.01	−0.12	−0.10	0.45	−0.19	0.39	NF-κB IL-6 and COX-2
Myricetin	−0.06	−0.18	0.28	0.32	−0.20	0.3	MAPK and NF-κB
Myricitrin	−0.02	−0.08	0.08	0.14	−0.06	0.38	TNF-α
Icariin	−0.41	−1.25	−0.75	−0.59	−0.34	−0.36	TNF- α, IL-1β and NO, NF-κB
Nobiletin	−0.13	−0.04	0.09	0	−0.22	0.11	TNF- α, IL-1β and NO, NF-κB
Tenuigenin	0.13	−0.22	−0.22	0.67	0.13	0.45	NLRP3 pro-IL-1β, and IL-1β
Tanshinone I	−0.34	−0.27	−0.09	−0.01	−0.62	−0.08	TNF-α, IL-10 IL-6, IL-1β
Salvianolic acid B	−0.66	−1.88	−1.52	−1.13	−0.54	−1.05	TNF-α, IL-1β, NO
Licochalcone E	−0.13	−0.20	−0.37	0.27	−0.23	−0.03	Nrf2/ARE-
Licochalcone A	−0.05	−0.03	−0.21	0.18	−0.25	0.1	ERK1/2 and NF-κB p65
Isobavachalcone	0.15	0.06	−0.17	0.44	0.02	0.38	NF-κB, TNF-α, IL-6, IL-1β, and IL-10
Macelignan	0	−0.04	−0.10	−0.04	−0.07	0.05	MAPKs and NF-kB, PPAR-γ
Ginsenoside Rg1	−1.34	−2.52	−2.34	−1.94	−0.92	−1.36	NF-κB and MAPK
Tripchlorolide	0.17	0.24	−0.41	0.51	0.36	0.7	TNF-α, IL-1β, NO, iNOS, PGE2, and COX-2
Triptolide	0.11	0.09	−0.43	0.4	0.24	0.86	NO, iNOS, TNF-α and IL-1β
Naringin	0.11	−0.40	−0.24	0.04	0.09	0.24	IL-1β, TNF-α
Cannabidiol	0.35	−0.14	−0.48	0.38	−0.19	0.33	TNF- α, IFN-γ, IL-17
Dimethyl fumarate	−1.22	−0.64	−1.57	−1.14	−1.11	−0.66	IGF-1, MRC1 TNF- α, IL-12
3H-1,2-dithiole-3-thione	−4.02	−4.01	−4.03	−4.03	−4.01	−3.67	IFN-γ and IL-17
Baicalin	−0.12	−0.18	0.19	0.17	−0.35	0.26	IFN-γ, IL-4 STAT/NF-κB
Matrine	0.21	−0.10	−0.60	−0.88	0.07	0.06	HSPB5, IL-1β
Oleanolic Acid	0.28	−0.06	−0.40	0.77	0.15	0.65	IFN-γ, TNF-α IL-10
Astragaloside IV	−1.17	−2.43	−2.13	−1.76	−0.86	−1.23	iNOS, IFN-γ, TNF-α and IL-6
Glycyrrhizin	−1.78	−3.09	−3.09	−2.36	−1.26	−1.93	TNF-α, IFN-γ IL-17A, IL-6 TGF-β1, IL-4
18β-Glycyrrhetinic Acid	0.24	−0.09	−0.59	0.79	0.21	0.7	MAPK, TNF- α and IL-1β
Carnosol	0.52	0.13	−0.26	0.51	−0.08	0.37	iNOS ARG-1 NO and TNF-α
Tanshinone IIA	−0.08	0.06	−0.23	0.22	−0.62	0.08	IL-17 and IL-23

**Table 5:** The Pharmacokinetics ADME Properties of the Neuroprotective Phytochemicals Used for AD, PD, and MS Treatments using SwissADME webserver.

Compounds Names	Molecular Weight	HB Donor	HB Acceptor	Log Po/w [WLOGP]	Log S [SILICO S-IT]	BBB Permeant	GI Absorption	P-gp Substrate	Rule of Five [ROF]
Curcumin	368.38 g/mol	2	6	3.15	−4.45	No	High	No	Yes: 0 violation
Aromatic-turmerone	216.32 g/mol	0	1	4.02	−4.45	Yes	High	No	Yes: 0 violation
Resveratrol	228.24 g/mol	3	3	2.76	−3.29	Yes	High	No	Yes: 0 violation
Pterostilbene	256.30 g/mol	1	3	3.36	−4.69	Yes	High	No	Yes: 0 violation
Sulforaphane	177.29 g/mol	0	2	2.11	−2.10	No	High	No	Yes: 0 violation
Epigallocatechin-3-gallate	458.37 g/mol	8	11	1.91	−2.50	No	Low	No	No; 2 violations: NorO > 10, NHorOH > 5
Andrographolide	350.45 g/mol	3	5	1.96	−2.69	No	High	Yes	Yes: 0 violation
Paeoniflorin	480.46 g/mol	5	11	−1.36	−1.15	No	Low	Yes	Yes; 1 violation: NorO > 10
β-caryophyllene	204.35 g/mol	0	0	4.73	−3.77	No	Low	No	Yes; 1 violation: MLOGP > 4.15
Oridonin	364.43 g/mol	4	6	0.38	−1.60	No	High	Yes	Yes: 0 violation
Dihydromyricetin	320.25 g/mol	6	8	0.57	−1.44	No	Low	No	Yes; 1 violation: NHorOH > 5
4-O-methylhonokiol	280.36 g/mol	1	2	4.52	−6.17	Yes	High	No	Yes: 0 violation
Silibinin	482.44 g/mol	5	10	1.71	−4.50	No	Low	No	Yes: 0 violation
Hesperidin	610.56 g/mol	8	15	−1.48	−0.58	No	Low	Yes	No; 3 violations: MW > 500, NorO > 10, NHorOH > 5
Triptolide	360.40 g/mol	1	6	1.1	−2.51	No	High	Yes	Yes: 0 violation
Eriodictyol	288.25 g/mol	4	6	1.89	−2.84	No	High	Yes	Yes: 0 violation
Xanthoceraside	1141.29 g/mol	12	23	0.26	0.2	No	Low	Yes	No; 3 violations: MW > 500, NorO > 10, NHorOH > 5
Piperlongumine	317.34 g/mol	0	5	1.55	−2.94	Yes	High	No	Yes: 0 violation
Esculentoside A	973.11 g/mol	11	20	−1.09	−0.08	No	Low	Yes	No; 3 violations: MW > 500, NorO > 10, NHorOH > 5
Quercetin	302.24 g/mol	5	7	1.99	−3.24	No	High	No	Yes: 0 violation
Apigenin	270.24 g/mol	3	5	2.58	−4.40	No	High	No	Yes: 0 violation
Capsaicin	305.41 g/mol	2	3	3.64	−4.87	Yes	High	No	Yes: 0 violation
α-asarone	208.25 g/mol	0	3	2.64	−3.26	Yes	High	No	Yes: 0 violation
Galangin	270.24 g/mol	3	5	2.58	−4.40	No	High	No	Yes: 0 violation
Biochanin A	284.26 g/mol	2	5	2.88	−5.10	No	High	No	Yes: 0 violation
Baicalein	270.24 g/mol	3	5	2.58	−4.40	No	High	No	Yes: 0 violation
Apocynin	166.17 g/mol	1	3	1.6	−2.28	Yes	High	No	Yes: 0 violation
α-Mangostin	410.46 g/mol	3	6	5.09	−6.14	No	High	No	Yes: 0 violation
Myricetin	318.24 g/mol	6	8	1.69	−2.66	No	Low	No	Yes; 1 violation: NHorOH > 5
Myricitrin	464.38 g/mol	8	12	0.19	−1.49	No	Low	No	No; 2 violations: NorO > 10, NHorOH > 5
Icariin	676.66 g/mol	8	15	0.07	−2.74	No	Low	Yes	No; 3 violations: MW > 500, NorO > 10, NHorOH > 5
Nobiletin	402.39 g/mol	0	8	3.51	−6.82	No	High	No	Yes: 0 violation
Tenuigenin	537.13 g/mol	4	6	5.49	−4.85	No	Low	Yes	No; 2 violations: MW > 500, MLOGP > 4.15
Tanshinone I	276.29 g/mol	0	3	4.1	−6.91	Yes	High	No	Yes; 0 violation
Salvianolic acid B	718.61 g/mol	9	16	2.9	−4.41	No	Low	No	No; 3 violations: MW > 500, NorO > 10, NHorOH > 5
Licochalcone E	338.40 g/mol	2	4	4.57	−5.17	Yes	High	No	Yes; 0 violation
Licochalcone A	338.40 g/mol	2	4	4.57	−5.17	Yes	High	No	Yes; 0 violation
Isobavachalcone	324.37 g/mol	3	4	4.1	−4.47	No	High	No	Yes; 0 violation
Macelignan	328.40 g/mol	1	4	4.19	−5.88	Yes	High	No	Yes; 0 violation
Ginsenoside Rg1	801.01 g/mol	10	40	1.12	−0.87	No	Low	Yes	No; 3 violations: MW > 500, NorO > 10, NHorOH > 5
Tripchlorolide	396.86 g/mol	2	6	1.3	−2.79	No	High	Yes	Yes; 0 violation
Triptolide	360.40 g/mol	1	6	1.1	−2.51	No	High	Yes	Yes; 0 violation
Naringin	580.53 g/mol	8	14	−1.49	−0.49	No	Low	Yes	No; 3 violations: MW > 500, NorO > 10, NHorOH > 5
Cannabidiol	314.46 g/mol	2	2	5.85	−5.41	Yes	High	No	Yes: 1 violation: MLOGP > 4.15
Dimethyl fumarate	144.13 g/mol	0	4	−0.11	−0.10	No	High	No	Yes; 0 violation
3H-1,2-dithiole-3-thione	134.24 g/mol	0	0	2.54	−1.43	No	High	No	Yes; 0 violation
Baicalin	270.24 g/mol	3	5	2.58	−4.40	No	High	No	Yes; 0 violation
Matrine	248.36 g/mol	0	2	1.11	−1.68	Yes	High	No	Yes; 0 violation
Oleanolic Acid	456.70 g/mol	2	3	7.23	−6.12	No	Low	No	Yes; 1 violation: MLOGP > 4.15
Astragaloside IV	784.97 g/mol	9	14	0.72	−1.11	No	Low	Yes	No; 3 violations: MW > 500, NorO > 10, NHorOH > 5
Glycyrrhizin	822.93 g/mol	8	16	2.25	−1.39	No	Low	Yes	No; 3 violations: MW > 500, NorO > 10, NHorOH > 5
18β-Glycyrrhetinic Acid	470.68 g/mol	2	4	6.41	−6.00	No	High	Yes	Yes; 1 violation: MLOGP > 4.15
Carnosol	330.42 g/mol	2	4	3.96	−4.45	Yes	High	Yes	Yes; 0 violation
Tanshinone IIA	294.34 g/mol	0	3	4.25	−6.71	Yes	High	Yes	Yes; 0 violation

**Table 6:** Cytochromes Inhibition Profile of the Neuroprotective Phytochemicals Used for AD, PD, and MS Treatments using SwissADME webserver.

Compound Names	CYP1A2	CYP2C19	CYP2C9	CYP2D6	CYP3A4
Curcumin	No	No	Yes	No	Yes
Aromatic turmerone	No	No	No	No	No
Resveratrol	Yes	No	Yes	No	Yes
Pterostilbene	Yes	Yes	Yes	Yes	No
Sulforaphane	No	No	No	No	No
Epigallocatechin-3-gallate	No	No	No	No	No
Andrographolide	No	No	No	No	No
Paeoniflorin	No	No	No	No	No
β-caryophyllene	No	Yes	Yes	No	No
Oridonin	No	No	No	No	No
Dihydromyricetin	No	No	No	No	No
4-O-methylhonokiol	Yes	Yes	Yes	Yes	Yes
Silibinin	No	No	No	No	Yes
Hesperidin	No	No	No	No	No
Triptolide	No	No	No	No	No
Eriodictyol	No	No	No	No	Yes
Xanthoceraside	No	No	No	No	No
Piperlongumine	No	No	No	No	No
Esculentoside A	No	No	No	No	No
Quercetin	Yes	No	No	Yes	Yes
Apigenin	Yes	No	No	Yes	Yes
Capsaicin	Yes	No	No	Yes	Yes
α-asarone	Yes	Yes	No	No	No
Galangin	Yes	No	No	Yes	Yes
Biochanin A	Yes	No	No	Yes	Yes
Baicalein	Yes	No	No	Yes	Yes
Apocynin	No	No	No	No	No
α-Mangostin	No	No	Yes	No	No
Myricetin	Yes	No	No	No	Yes
Myricitrin	No	No	No	No	No
Icariin	No	No	No	No	No
Nobiletin	No	No	Yes	No	Yes
Tenuigenin	No	No	No	No	No
Tanshinone I	Yes	Yes	No	No	Yes
Salvianolic acid B	No	No	No	No	No
Licochalcone E	Yes	No	Yes	No	Yes
Licochalcone A	Yes	No	Yes	No	Yes
Isobavachalcone	Yes	No	Yes	No	Yes
Macelignan	No	Yes	Yes	Yes	No
Ginsenoside Rg1	No	No	No	No	No
Tripchlorolide	No	No	No	No	No
Triptolide	No	No	No	No	No
Naringin	No	No	No	No	No
Cannabidiol	No	Yes	Yes	Yes	Yes
Dimethyl fumarate	No	No	No	No	No
3H-1,2-dithiole-3-thione	No	No	No	No	No
Baicalin	Yes	No	No	Yes	Yes
Matrine	No	No	No	No	No
Oleanolic Acid	No	No	No	No	No
Astragaloside IV	No	No	No	No	No
Glycyrrhizin	No	No	No	No	No
18β-Glycyrrhetinic Acid	No	No	No	No	No
Carnosol	No	No	Yes	No	No
Tanshinone IIA	Yes	Yes	Yes	Yes	Yes

**Table 7:** The Toxicity Profiles of the Neuroprotective Phytochemicals Used for AD, PD, and MS Treatments using ProTox-II online Tool.

Compound Names	Predicted Toxicity Class	Predicted LD_50_ [mg/kg]	Organ toxicity/ Toxicity endpoints	Probability
Curcumin	4	2000	Hepatotoxicity	0.61
			Carcinogenicity	0.84
			Mutagenicity	0.88
			Immunotoxicity	0.92
Aromatic-turmerone	4	2000	Hepatotoxicity	0.59
			Carcinogenicity	0.64
			Mutagenicity	0.93
			Immunotoxicity	0.99
Resveratrol	4	1560	Hepatotoxicity	0.74
			Carcinogenicity	0.71
			Mutagenicity	0.92
			Immunotoxicity	0.86
Pterostilbene	4	1560	Hepatotoxicity	0.67
			Carcinogenicity	0.61
			Mutagenicity	0.81
			Immunotoxicity	0.65
Sulforaphane	4	1000	Hepatotoxicity	0.69
			Carcinogenicity	0.62
			Mutagenicity	0.63
			Immunotoxicity	0.99
Epigallocatechin-3-gallate	4	1000	Hepatotoxicity	0.70
			Carcinogenicity	0.54
			Mutagenicity	0.70
			Immunotoxicity	0.89
Andrographolide	4	1890	Hepatotoxicity	0.93
			Carcinogenicity	0.83
			Mutagenicity	0.71
			Immunotoxicity	0.82
Paeoniflorin	5	4000	Hepatotoxicity	0.90
			Carcinogenicity	0.85
			Mutagenicity	0.61
			Immunotoxicity	0.86
β-caryophyllene	5	5300	Hepatotoxicity	0.80
			Carcinogenicity	0.70
			Mutagenicity	0.95
			Immunotoxicity	0.54
Oridonin	3	120	Hepatotoxicity	0.86
			Carcinogenicity	0.69
			Mutagenicity	0.56
			Immunotoxicity	0.98
Dihydromyricetin	4	2000	Hepatotoxicity	0.69
			Carcinogenicity	0.68
			Mutagenicity	0.51
			Immunotoxicity	0.59
4-O-methylhonokiol	4	1649	Hepatotoxicity	0.71
			Carcinogenicity	0.64
			Mutagenicity	0.89
			Immunotoxicity	0.50
Silibinin	4	2000	Hepatotoxicity	0.78
			Carcinogenicity	0.72
			Mutagenicity	0.69
			Immunotoxicity	0.97
Hesperidin	6	12,000	Hepatotoxicity	0.81
			Carcinogenicity	0.93
			Mutagenicity	0.90
			Immunotoxicity	0.99
Triptolide	1	4	Hepatotoxicity	0.88
			Carcinogenicity	0.58
			Mutagenicity	0.75
			Immunotoxicity	0.97
Eriodictyol	4	2000	Hepatotoxicity	0.67
			Carcinogenicity	0.57
			Mutagenicity	0.59
			Immunotoxicity	0.71
Xanthoceraside	4	590	Hepatotoxicity	0.94
			Carcinogenicity	0.68
			Mutagenicity	0.92
			Immunotoxicity	0.99
Piperlongumine	4	1180	Hepatotoxicity	0.79
			Carcinogenicity	0.52
			Mutagenicity	0.69
			Immunotoxicity	0.99
Esculentoside A	5	4000	Hepatotoxicity	0.95
			Carcinogenicity	0.73
			Mutagenicity	0.96
			Immunotoxicity	0.99
Quercetin	3	159	Hepatotoxicity	0.69
			Carcinogenicity	0.68
			Mutagenicity	0.51
			Immunotoxicity	0.87
Apigenin	5	2500	Hepatotoxicity	0.86
			Carcinogenicity	0.62
			Mutagenicity	0.57
			Immunotoxicity	0.99
Capsaicin	2	47	Hepatotoxicity	0.88
			Carcinogenicity	0.71
			Mutagenicity	0.51
			Immunotoxicity	0.86
α-asarone	4	418	Hepatotoxicity	0.63
			Carcinogenicity	0.56
			Mutagenicity	0.92
			Immunotoxicity	0.67
			Immunotoxicity	0.99
Galangin	5	3919	Hepatotoxicity	0.68
			Carcinogenicity	0.72
			Mutagenicity	0.52
			Immunotoxicity	0.97
Biochanin A	5	2500	Hepatotoxicity	0.73
			Carcinogenicity	0.65
			Mutagenicity	0.94
			Immunotoxicity	0.75
Baicalein	5	3919	Hepatotoxicity	0.69
			Carcinogenicity	0.68
			Mutagenicity	0.51
			Immunotoxicity	0.99
Apocynin	6	9000	Hepatotoxicity	0.52
			Carcinogenicity	0.57
			Mutagenicity	0.99
			Immunotoxicity	0.78
α-Mangostin	4	1500	Hepatotoxicity	0.70
			Carcinogenicity	0.69
			Mutagenicity	0.53
			Immunotoxicity	0.84
Myricetin	3	159	Hepatotoxicity	0.69
			Carcinogenicity	0.68
			Mutagenicity	0.51
			Immunotoxicity	0.86
Myricitrin	5	5000	Hepatotoxicity	0.73
			Carcinogenicity	0.50
			Mutagenicity	0.71
			Immunotoxicity	0.98
Icariin	5	5000	Hepatotoxicity	0.74
			Carcinogenicity	0.83
			Mutagenicity	0.70
			Immunotoxicity	0.98
Nobiletin	5	5000	Hepatotoxicity	0.69
			Carcinogenicity	0.53
			Mutagenicity	0.69
			Immunotoxicity	0.51
Tenuigenin	6	6176	Hepatotoxicity	0.94
			Carcinogenicity	0.51
			Mutagenicity	0.86
			Immunotoxicity	0.86
Tanshinone I	4	1655	Hepatotoxicity	0.63
			Carcinogenicity	0.51
			Mutagenicity	0.55
			Immunotoxicity	0.66
Salvianolic acid B	2	25	Hepatotoxicity	0.64
			Carcinogenicity	0.60
			Mutagenicity	0.55
			Immunotoxicity	0.97
Licochalcone E	4	1000	Hepatotoxicity	0.51
			Carcinogenicity	0.67
			Mutagenicity	0.68
			Immunotoxicity	0.92
Licochalcone A	4	1000	Hepatotoxicity	0.62
			Carcinogenicity	0.60
			Mutagenicity	0.79
			Immunotoxicity	0.76
Isobavachalcone	4	1000	Hepatotoxicity	0.64
			Carcinogenicity	0.72
			Mutagenicity	0.76
			Immunotoxicity	0.97
Macelignan	5	2260	Hepatotoxicity	0.75
			Carcinogenicity	0.50
			Mutagenicity	0.51
			Immunotoxicity	0.97
Ginsenoside Rg1	5	4000	Hepatotoxicity	0.94
			Carcinogenicity	0.74
			Mutagenicity	0.91
			Immunotoxicity	0.88
Tripchlorolide	1	4	Hepatotoxicity	0.88
			Carcinogenicity	0.60
			Mutagenicity	0.75
			Immunotoxicity	0.99
Triptolide	1	4	Hepatotoxicity	0.88
			Carcinogenicity	0.58
			Mutagenicity	0.75
			Immunotoxicity	0.97
Naringin	5	2300	Hepatotoxicity	0.81
			Carcinogenicity	0.90
			Mutagenicity	0.73
			Immunotoxicity	0.99
Cannabidiol	4	500	Hepatotoxicity	0.79
			Carcinogenicity	0.66
			Mutagenicity	0.85
			Immunotoxicity	0.93
Dimethyl fumarate	3	62	Hepatotoxicity	0.80
			Carcinogenicity	0.74
			Mutagenicity	0.71
			Immunotoxicity	0.99
3H-1,2-dithiole-3-thione	4	1480	Hepatotoxicity	0.68
			Carcinogenicity	0.50
			Mutagenicity	0.81
			Immunotoxicity	0.99
Baicalin	5	3919	Hepatotoxicity	0.69
			Carcinogenicity	0.68
			Mutagenicity	0.51
			Immunotoxicity	0.99
Matrine	3	243	Hepatotoxicity	0.92
			Carcinogenicity	0.68
			Mutagenicity	0.77
			Immunotoxicity	0.96
Oleanolic Acid	4	2000	Hepatotoxicity	0.52
			Carcinogenicity	0.57
			Mutagenicity	0.85
			Immunotoxicity	0.79
Astragaloside IV	6	23,000	Hepatotoxicity	0.92
			Carcinogenicity	0.74
			Mutagenicity	0.67
			Immunotoxicity	0.99
Glycyrrhizin	4	1750	Hepatotoxicity	0.88
			Carcinogenicity	0.61
			Mutagenicity	0.96
			Immunotoxicity	0.99
18β-Glycyrrhetinic Acid	4	560	Hepatotoxicity	0.69
			Carcinogenicity	0.55
			Mutagenicity	0.90
			Immunotoxicity	0.94
Carnosol	4	1500	Hepatotoxicity	0.76
			Carcinogenicity	0.62
			Mutagenicity	0.88
			Immunotoxicity	0.99
Tanshinone IIA	4	1230	Hepatotoxicity	0.71
			Carcinogenicity	0.56
			Mutagenicity	0.70
			Immunotoxicity	0.80

Class 1 Fatal if swallowed [LD_50_ ≤ 5], Class 2 Fatal if swallowed [5 < LD_50_ ≤ 50], Class 3 Toxic if swallowed [50 < LD_50_ ≤ 300], Class 4 Harmful if swallowed [300 < LD_50_ ≤ 2000], Class 5 It may be harmful if swallowed [2000 < LD_50_ ≤ 5000],Class 6 Non-toxic [LD_50_ > 5000].

## Abbreviation

AD	Alzheimer’s disease
ADE	amyloid degrading enzymes
AEP	asparagine endopeptidase
AKT/GSK	3β: protein kinase B/glycogen synthase kinase-3beta
AMPK/SIRT1	Adenosine monophosphate-activated protein kinase [AMPK]/NAD-dependent deacetylase sirtuin-1 [SIRT1]
AP-1	activator protein-1
ARE	antioxidant response element
ARG1	Arginase-1
Aβ	amyloid-beta
BBB	blood-brain barrier penetration
CB2R	cannabinoid receptor 2
CD206	macrophage mannose receptor
CNTFRα	ciliary neurotrophic factor receptor alpha
COX2	Cyclooxygenase-2
CSFs	colony-stimulating factors
CYP450	cytochrome P450
ERK_1/2_	Extracellular signal-regulated kinase
GABA-B	γ-aminobutyric acid type B
GPCRs	G protein-coupled receptors
HO-1	heme oxygenase-1
IDE	insulin-degrading enzyme
IFNs	interferons
IKK	IκB kinase
IL	interleukins
INF-γ/LPS	interferon-gamma combined with lipopolysaccharide
iNOS	Inducible nitric oxide synthase
IκB	NF-κB inhibitor
JAK-STAT	Janus kinase signal transducer and activator of transcription
JNK	c-Jun N-terminal kinase
logS	high solubility
LOO-CV	leave-one-out cross-validation
LPS	Lipopolysaccharide
MAPK	mitogen-activated protein kinase
MCP-1	monocyte chemoattractant protein-1
MS	Multiple sclerosis
mTOR	mammalian target of rapamycin
MW	molecular weight
NDs	neurodegeneration diseases
NEP	neprilysin
NF-κB	nuclear factor-kappa-B
NFT	neurofibrillary tangles
NLRP3	NLR family pyrin domain containing 3
NLRs	nucleotide-binding oligomerization domain [nod]-like receptors
NO	nitric oxide
NOX2	nicotinamide adenine dinucleotide phosphate [NADPH] oxidase-2
Nrf2	nuclear factor erythroid 2-related factor 2
P-gp	P-glycoprotein
Pa:Pi	active, inactive ratio
PASS	predict the activity spectra of substances
PD	Parkinson’s disease
PGE2	prostaglandin E2
PI3K/Akt	phosphatidylinositol-3-Kinase and Protein/Kinase B
PIKKs	phosphatidylinositol 3-kinase-related kinase
PPARs	Peroxisome proliferator-activated receptors
PRRs	pattern-recognition receptors
ROS	reactive oxygen species
SMILES	simplified molecular-input line-entry system
SRC	non-receptor protein tyrosine kinase
STATs	signal transducer and activator of transcription
TGF-β	transforming growth factor-beta
TLRs	toll-like receptors
TNF-α	tumor necrosis factor-α
TREMs	triggering receptor expressed on myeloid cells
TRP	potential transient receptors
WBC	white blood cells
α-SYN	alpha-synuclein
